# Microbial community shifts during salt mitigation treatments of historic buildings using mineral poultices: a long-term monitoring of salt and associated biofilms

**DOI:** 10.3389/fmicb.2025.1603289

**Published:** 2025-05-22

**Authors:** Johannes Tichy, Beate Sipek, Martin Ortbauer, Lukas Fürnwein, Monika Waldherr, Alexandra Graf, Katja Sterflinger, Guadalupe Piñar

**Affiliations:** ^1^Institute for Natural Sciences and Technology in the Art, Academy of Fine Arts Vienna, Vienna, Austria; ^2^Institute for Conservation – Restoration, Academy of Fine Arts Vienna, Vienna, Austria; ^3^Department of Applied Life Sciences/Bioengineering/Bioinformatics, FH Campus Wien, Vienna, Austria

**Keywords:** salt-weathering, mineral poultices, pink biofilms, archaea, bacteria, long-term monitoring, metataxonomics

## Abstract

Increased heavy rainfall followed by periods of drought due to climate change is leading to more frequent salt-crystallization cycles. This not only leads to increased salt-weathering on architectural surfaces of cultural heritage monuments, but also creates an ideal ecological niche for the formation of biofilms by salt-loving microorganisms. These biofilms, characterized by a distinctive pink coloration, cause additional esthetic alterations to affected surfaces. In this study, mineral poultices prepared with different clay minerals (sepiolite, kaolinite and vermiculite) were developed and tested for a long-term (1 year) application on salt-weathered surfaces, thus contributing to their preservation. The poultices were tested on the surfaces of two historic buildings: the St. Virgil’s Chapel in Vienna and the Mauerbach Charterhouse in Lower Austria, both showing salt efflorescence and a uniform pink biofilm. First, the poultices were tested to evaluate their salt retention capacity, salt-weathering resistance and processability. The retention properties of the poultices were examined by measuring their salt content throughout the treatment using high performance liquid chromatography (HPLC) and continuous flow analysis (CFA). Salt content was also measured on the wall surfaces before and after treatment. Second, the effect of the desalination treatments on salt-associated pink biofilms was also evaluated. The shifts within the biofilm communities during and after the treatment were monitored by qPCR and long-read archaeal- and bacterial-16S rRNA amplicon analysis using the Nanopore sequencing technology. The results demonstrate that both the selected clay minerals and the salt composition in the treated areas significantly influenced the salt storage capacity of the poultices and their resistance to salt weathering. Fluctuations in salt load and ionic composition during and after treatment affected biofilm composition, with bacterial communities proving more sensitive than archaea to these changes. Both qPCR and metataxonomic results show that the effects of the poultices on the colonizing biofilms depend not only on the composition of their microbial members, but also on external abiotic factors such as the chemical composition and concentration of the salt mixtures on the surfaces. In addition, the biodiversity within the biofilms shows to be affected differently depending on the mineral clay used.

## 1 Introduction

Time and climate change are two uncontrollable factors that challenge the preservation of cultural heritage. Particularly in Central Europe, increasing heavy rainfalls, alternating with periods of intense drought, promotes the formation of salt efflorescence, which endangers cultural heritage monuments (Leissner et al., 2015). This phenomenon not only creates new ecological niches for halophilic microorganisms, facilitating microbial growth on monument surfaces, but also complicates restoration efforts in maintaining the integrity of inorganic surfaces. It has been reported that the process of microbial colonization in built cultural heritage heavily contaminated by salt very often leads to the phenomenon of pinkish discolouration (Imperi et al., 2007; Piñar et al., 2014; Tichy et al., 2023). This discolouration is due to halophilic/halotolerant microbial communities producing carotenoid pigments, mostly bacterioruberin (BR), which are dispersed into the cultural heritage environment, causing the characteristic pinkish appearance (Imperi et al., 2007; Ettenauer et al., 2014; Tescari et al., 2018b; Cojoc et al., 2019; Basile et al., 2025). The main players in the discolouration phenomenon are halophilic bacteria (Saiz-Jimenez and Laiz, 2000; Schabereiter-Gurtner et al., 2001) and archaea (Rölleke et al., 1998; Piñar et al., 2001), usually embedded into a biofilm located either on top of the inorganic surface or within the salt efflorescence itself. Halophilic communities, besides causing esthetic damage through the pigment production, may also contribute to the damage of architectural surfaces by changing the inorganic surface properties through the process of biodegradation (Schröer et al., 2021).

Salt crystallization exerts a destructive force on construction materials (Arnold and Zehnder, 1991; Charola, 2000). To prevent further degradation of affected cultural heritage surfaces, remediation or mitigation of harmful salts is required (Charola and Bläuer, 2015). For the removal of salt efflorescence, a classical approach includes the application of poultices (Verges-Belmin and Siedel, 2005; Lubelli and Van Hees, 2010; Lenz, 2017), which function based on diffusion and advection principles (Pel et al., 2010). While the physical effects of desalination and stabilization treatments on architectural surfaces are well-documented, even over the long term (Zehnder, 2007; Heritage et al., 2013; Laue et al., 2021), less attention has been given to their biological impact. Few studies have monitored microbial communities over extended periods following salt reduction or bio-cleaning treatments (Bosch-Roig et al., 2015; Pinna et al., 2018; Ranalli and Zanardini, 2021; Pavlović et al., 2022). Long-term microbial monitoring after salt reduction is essential, as variations in salt composition and ion concentration shifts can influence microbial communities on deteriorated surfaces (Adamiak et al., 2015; Tichy et al., 2023). Additionally, for inorganic surfaces such as stone or mortar, it is difficult to distinguish between damage caused by either physical and/or chemical processes and that caused by microbiological activity (Steiger et al., 2011). Therefore, when assessing treatment effectiveness, both salt composition and concentration changes, as well as their impact on microbial communities, must be monitored before, during, and after cleaning, desalination, or consolidation treatments.

In this study, a long-term desalination treatment was applied to salt-contaminated surfaces at two historic sites: St. Virgil’s Chapel and the Charterhouse Mauerbach. Both locations have been comprehensively analyzed for salt composition and associated microbial biofilms (Tichy et al., 2023). To mitigate salt-induced damage, we applied mineral poultices composed of different clay minerals—sepiolite, kaolinite, and vermiculite —for 1 year and monitored the treatment over time. These minerals were selected due to their distinct physicochemical properties relevant for salt extraction (Bergaya and Lagaly, 2013). Sepiolite, a fibrous clay with high specific surface area and porosity, facilitates capillary transport and salt uptake. Kaolinite, a plate-like clay with lower cation exchange capacity, offers dimensional stability and ease of handling. Vermiculite, with its high ion-exchange capacity and swelling behavior, can enhance ion mobility and retention. Such properties suggest that these clays can support both effective salt removal and adaptability to surface conditions in heritage conservation applications. In addition, natural clays, especially kaolinite and vermiculite used in this study, show antimicrobial properties (Williams and Haydel, 2010; Londono et al., 2017) and could be functionalised for the release of biocidal agents (Gallo et al., 2020; Pastor et al., 2020) in order to obtain better control of the microbiomes associated with salt-weathered surfaces in heritage buildings. However, the raw materials used in this study were not modified with biocides. Similar clay materials have been used in desalination studies targeting construction materials and artworks (Verges-Belmin and Siedel, 2005; Lubelli and Van Hees, 2010), but a comparative evaluation of different clays over long treatment periods remains limited. Therefore, to assess their potential for a long-term application, this study tested the salt retention capacity of each poultice, its resistance to salt weathering and its handling properties. In addition, the treatment effects on surface ion concentrations and on the colonizing biofilms were monitored by metataxonomic profiling and quantitative PCR.

## 2 Materials and methods

### 2.1 Description of the locations of treated surfaces

**Figure 1:**
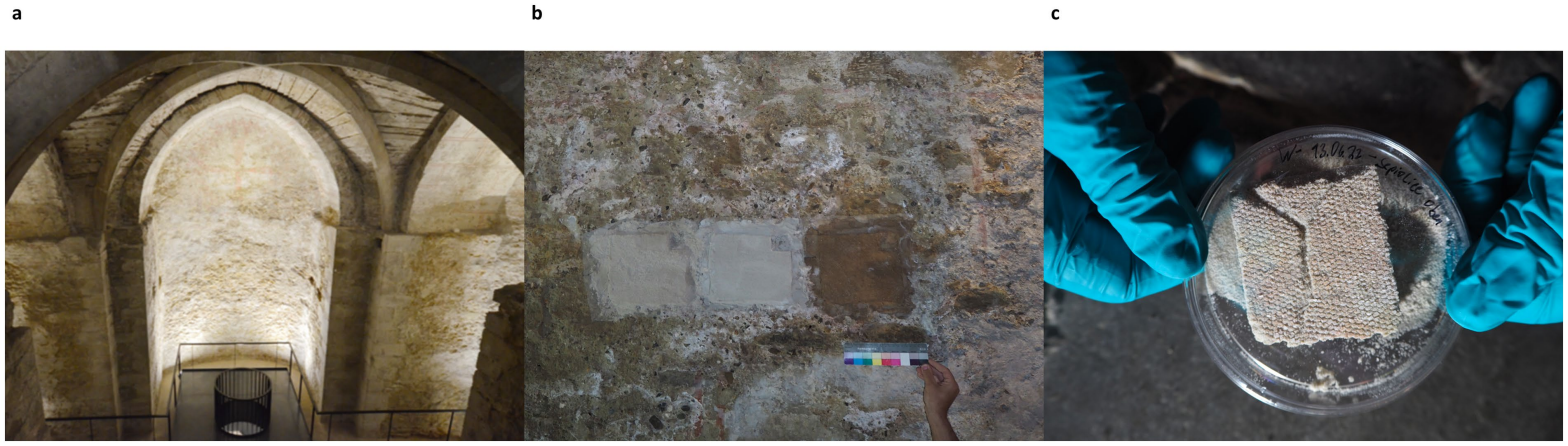
Example of the application of mineral poultices in St. Virgil’s Chapel: **(a)** view from above of St. Virgil’s Chapel; **(b)** application of mineral poultices [from left to right: sepiolite; kaolinite; vermiculite]; **(c)** aseptically sampling procedure of poultices [5 × 5 cm^3^].

The treatment was applied to two historic buildings. First of all, the Chapel of St. Virgil (1,220/30 AD) Vienna (Austria), has been the subject of numerous microbial investigations (Ripka et al., 2006; Piñar et al., 2009; Ettenauer et al., 2010) (Figure 1a). A more recent study also included climatic and salt chemistry analyses (Tichy et al., 2023). The chapel, located underground, exhibits severe salt damage, primarily due to halite (NaCl) efflorescence. This results from water infiltration following heavy rainfalls, which dissolves and transports de-icing salts used in winter. The surface of the rendering is rough, highly porous and structurally weakened due to progressive weathering. The poultices were tested on a previously investigated vault, designated hereafter as V2 (Tichy et al., 2023).

The second site, the Charterhouse in Mauerbach (Lower Austria), is a former monastery built in 1314, also previously studied (Tichy et al., 2023). Here, water ingress is caused by capillary forces (rising damp). The investigated plastered surfaces of the walls are smooth with negligible porosity. Poultices were applied in two rooms: M4, which showed no visible salt efflorescence but contained gypsum, and M6, which exhibited extensive salt efflorescence, primarily thenardite (Na₂SO₄) (Tichy et al., 2023).

### 2.2 Poultice compositions and application

Three clay minerals with distinct properties were selected for the preparation of mineral poultices for desalination tests in the two historical sites. The selected minerals were kaolinite, sepiolite and vermiculite. The poultices remained on the surfaces for a total period of 1 year. The clay minerals were combined with quartz sand and perlite as additives. The addition of perlite serves the purpose of reducing the overall weight of the mixture, which is an important factor for the application on salt-damaged plaster structures. The mixtures consisted of one volume part of each clay mineral, one volume part of perlite, and two to three volume parts of quartz sand, with the addition of 1–1.5 volume parts of water, to create a mixture that was easily applicable. The proportion of quartz sand and water was dependent upon the properties of the clay mineral.

Three test areas (each 400 cm^2^) were selected in each of the test locations of the two sites (Figure 1b). The poultices were applied in a 1 cm-thick layer over a polyamide mesh (2 mm mesh size) using a spatula. The intermediate layer of polyamide was inserted to facilitate the removal of the poultice and thus protect the historic surfaces.

### 2.3 Sampling

Sampling of the different applied poultices was carried out at 1, 6, and 12 months of treatment. At each time point, for each location and poultice type, a 5 × 5 × 1 cm section of the poultice was aseptically removed from the wall, placed into a petri dish (Figure 1c) and transported on ice to the laboratory. Samples were stored at −20°C until further biological analysis. Before analysis, the poultices were homogenized using a sterile scalpel to obtain a fine granular mass.

Sampling of the walls after 12 months of treatment with the different poultices, hereafter referred to as “treated” (T) samples, was following the same procedure described for untreated walls (“NT” samples) in Tichy et al. (2023). Briefly, the treated pink biofilm surface of 5 × 5 cm was scratched with a sterile scalpel, collecting the fine obtained powder in a falcon tube, transported on ice to the laboratory and stored at −20°C until further analysis.

### 2.4 DNA extraction, library preparation and sequencing with Nanopore sequencing platform

The DNA extraction, from poultices and wall surfaces, was done in triplicate for each sample with the FastDNA Spin Kit for soil (MP Biomedicals, Illkirch, France), as explained in Tichy et al. (2023). DNA extracts of the poultices were pooled following a volume reduction through a vacuum concentrator at 25°C (Savant SpeedVac DNA 130, Thermo Fisher Scientific, Waltham, United States).

Full-length archaeal and bacterial 16S rRNA amplicons were obtained through a first PCR round, using the primers 27F/1942R for bacteria and SSU1ArF/SSU1000ArR for archaea, as explained by Tichy et al. (2023) with the modification that only 30 cycles were used in this study for the archaeal amplification. Afterwards the amplification products were cleaned up with NucleoSpin Gel and PCR Clean-up XS (Ref: 740611, MACHEREY-NAGEL, Düren/Germany) according to the instructions made by the manufacturer.

Then long-amplicon bacterial 16S rRNA gene library preparation and sequencing was done according to the protocol of the Oxford Nanopore Barcoding Kit (SQK-PBK004, nested-pcr-protocol-FFP_9038_v108_rev S_14Aug2019-minion-1) with all extracted samples. The used primers were 27F and 1492R configured with an adapter predefined by Oxford Nanopore Technologies (Forward primer: 5′-TTTCTGTTGGTG CTGATATTGC-27F-3′; Reverse primer: 5′-ACTTGCCTGTCGCTCTATCTTC-1492R-3′) and synthesized by Eurofins Genomics. Moreover, this was also done identically for the archaeal amplicons with the primers SSU1ArF/SSU1000ArR including the Oxford Nanopore Technologies specific adapters (Forward primer: 5′-TTTCTGTTGGTG CTGATATTGC-SSU1ArF-3′; Reverse primer: 5′-ACTTGCCTGT CGCTCTATCTTC-SSU1000ArR-3′) also synthesized by Eurofins Genomics. Detailed description of the Sequencing-PCR setup, primers, used enzymes and devices can be found in Tichy et al. (2023).

Finally, the barcoded sequences were pooled (bacterial- and archaeal-amplicons separately) in desired ratios to a total molar concentration ranging between 50 and 100 fmol in 10 μL and ligated to the provided sequencing adapters. The prepared libraries were loaded onto the MinION flow cell FLO-MIN 106D R9 Version (Oxford Nanopore Technologies, Oxford, UK). Sequencing was performed on the MinION Mk1C device for 48 h.

### 2.5 Bioinformatic analyses

FAST5 files, were basecalled using the Guppy basecalling software (Oxford Nanopore Technologies, Oxford, UK, version 5.0.11 + 2b6dbff) with the high accuracy (HAC) model. FastQC (v0.12.1) (Andrews et al., 2010) and Nanostat (1.6.0) (De Coster et al., 2018) were used for quality control. Adapter, barcode, and primer sequences were removed, and chimeric sequences were split using Porechop (0.2.4) (Wick et al., 2017). A head and tail crop of 40 bases and filtering for a mean quality score of 9 was done with NanoFilt (2.8.0). Additionally, a length filtering was conducted for bacterial and archaeal reads (1,000–1,600 bases for *bacteria*, 700–1,200 bases for *archaea*, according to the expected length of the PCR product).

Emu (v3.4.4) (Curry et al., 2022) was used to perform metataxonomic classifications with a combination of rrnDB v5.6 (Stoddard et al., 2015) and NCBI 16S RefSeq from 17 September 2020 (O’Leary et al., 2016). The resulting database comprises 49,301 sequences from 17,555 unique bacterial and archaeal species.

R version 4.3.2 and the packages pheatmap (version 1.0.12) (Kolde, 2010), taxonomizer (version 0.10.7) (Sherrill-Mix, 2017), phyloseq (McMurdie and Holmes, 2013), vegan (version 2.7-0) (Oksanen et al., 2025), microbiome (Lahti and Shetty, 2017), ggplot2 (Wickham, 2016), and tidyverse (Wickham et al., 2019) were used to perform relative abundance analysis, taxonomic clustering and the different diversity indices.

The data for the non-treated samples are derived from NCBI BioProject PRJNA909753 (Tichy et al., 2023). Data derived from this study are available under the NCBI BioProject accession Number PRJNA1242577.

### 2.6 Quantitative PCR analyses

To quantify the bacterial and archaeal load in the wall surfaces before and after the treatment, qPCR analyses were performed using Bio-Rad iTaq Universal SYBR Green Supermix. The total reaction volume was 10 μL. Each reaction mixture consisted of 1 μL template DNA, 1 μL primer mix, 5 μL iTaq Universal SYBR Green Supermix and 3 μL nuclease free water. The specific primers used for archaeal detection were ARC344F (ACGGGGYGCAGCAGGCGCGA) and ARC744R (CCSGGGTATCTAATCC), while bacterial quantification was performed with the BAC338F (ACTCCTACGGGAGGCAG) and BAC805R (GACTACCAGGGTATCTATCC) primers, synthesized by Eurofins Genomics. Each sample was performed in triplicate. Thermal cycling conditions followed the iTaq Universal SYBR Green Supermix protocol recommendations and Fluorescent Quantitative Detection System (Hangzhou Bioer technology/Shanghai). The PCR parameters included: 4 min denaturation at 95°C, followed by 40 cycles consisting of 5 s. denaturation at 95°C, 30 s. primer annealing and extension at 60°C. For the statistical analysis of the qPCR results between the sampling locations, the statistics program XLSTAT 2023.1.6.1410 was used [Two-sample *t*-test, significance level α = 0.05]. The ∆Ct (delta cycle threshold) values were calculated as follows: the mean Ct values of the treated samples (T) minus the mean values of the non-treated samples (NT).

### 2.7 Salt analysis

For the quantitative analyses of the salt composition High Performance Liquid Chromatography (HPLC) and continuous flow analysis (CFA) were used. The surface of 25 cm^2^ of treated wall surfaces (T) and 25 cm^3^ of the mineral poultices were analyzed. Non-treated walls (NT) were previously examined (Tichy et al., 2023). The collected material (poultices and historic surfaces) from the standardized surface area of 25 cm^2^ was weighed before and after drying at 60°C and dissolved in 60 mL demineralised water (according to VDE 0510). The samples were shaken for 3 min. and the non-dissolved particles were allowed to settle for 60 min. Subsequently, 20 mL of supernatant, free of non-dissolved particles, was transferred in a new 50 mL Falcon-Tube and stored at 7°C until analysis. Nitrate detection followed the DIN EN ISO 13395 procedure, involving the reduction of nitrate to nitrite using cadmium, followed by photometric determination after a nitration reaction with 2,6-dimethylphenol in a sulfuric/phosphoric acid solution via CFA.

Sulfate and Chloride ions were detected according DIN EN ISO 10304-1, using HPLC with ion separation and a conductivity detector (IC). Sodium, potassium, calcium, and magnesium ions were quantified in accordance with DIN EN 14911 using HPLC coupled with a conductivity detector. Details of the analytical equipment are provided in the Supplementary Table S1 (Tichy et al., 2023). To standardize the comparison of salt amounts detected on surfaces (cm^2^) and those extracted within the mineral poultices (cm^3^), a conversion factor of 1:1 was applied, and all values are reported in μg/cm^2^ aligning with the WTA Guideline (Wissenschaftlich-Technische Arbeitsgemeinschaft für Bauwerkserhaltung und Denkmalpflege, 2019), based on the mass of the detected ionic species. The total retention capacity of ionic species in the mineral poultices was calculated empirically as follows: all cations were normalized to sodium ions (Na^+^ eq.), and all anions to chloride ions (Cl^−^ eq.), based on molar amounts. The total sum of anion and cation equivalents was divided by two (simplified ion balancing through the formation of mean values) and multiplied by the molar mass of sodium chloride (NaCl), providing the empirical total salt retention capacity of the mineral poultice in μg/cm^2^.

## 3 Results

### 3.1 Effectiveness of treatment using different mineral poultices

**Figure 2:**
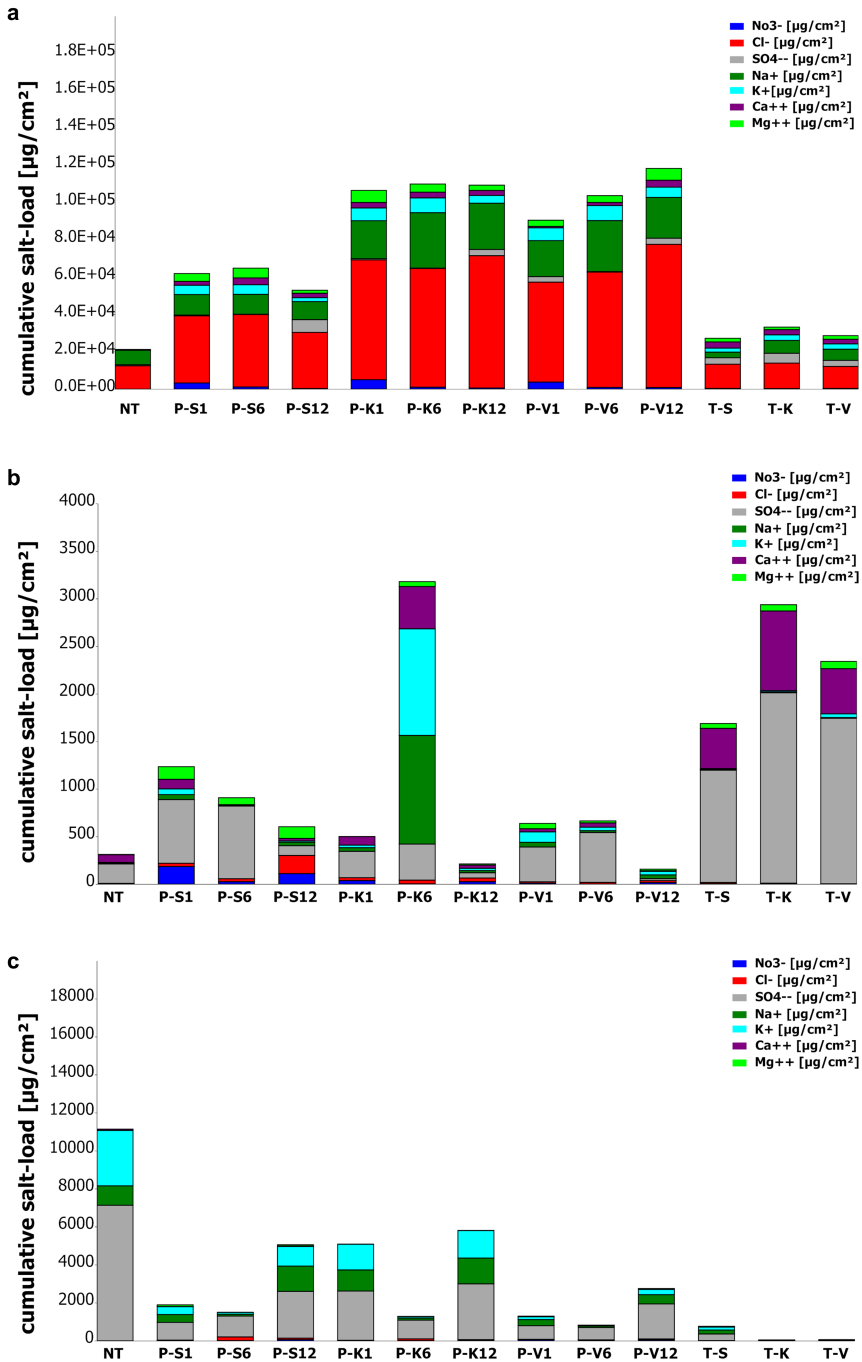
Quantitative salt analysis of sampling locations **(a)** V2; **(b)** M4, **(c)** M6 during the application of the poultices (Poultice [P]; Sepiolite [S]; Kaolinite [K]; Vermiculite [V]; non-treated [NT], after the treatment [T + S/K/V]) within the time intervals after 1, 6, and 12 months. Colors correspond to measured ions (μg/cm^2^) [sodium (dark-green); potassium (turquoise); magnesium (light-green); calcium (purple); sulfate (gray); nitrate (blue) and chloride (red)].

The effectiveness of the three tested poultices (sepiolite, kaolinite, and vermiculite) was evaluated in terms of their salt storage capacity and resistance to salt weathering. The salt concentration in the test areas was measured before and after treatment to determine the potential of the poultices to reduce salt content on the surfaces (Figure 2; Supplementary Table S1).

The poultices were tested in two historic buildings, each presenting distinct salt compositions and concentrations (see methods section and Tichy et al., 2023). At the St. Virgil’s Chapel, one of the vaults (V2) was examined, while at the Charterhouse of Mauerbach, two rooms (M4 and M6) were investigated (see methods section and Tichy et al., 2023). The three poultices exhibited different behaviors regarding salt retention, extraction efficiency, and resistance to salt weathering at various sampling points.

#### 3.1.1 St. Virgil Chapel

At the beginning of the monitoring campaign the surfaces of the St. Virgil Chapel were predominantly affected by halite (NaCl) salt efflorescence, with sulfates present to lesser extent, as reported previously (Tichy et al., 2023). Results show that all poultice mixtures applied in the chapel of St. Virgil extracted and retained sodium (with a max. in kaolinite after 6 months: 29716.8 μg/cm^2^) and chloride ions (max. in vermiculite after 12 months: 76798.2 μg/cm^2^) (Supplementary Table S1) during the application period. Additionally, potassium, calcium, magnesium, nitrate, and sulfate accumulated in all poultices (Figure 2a). Notably, all poultices exhibited significant nitrate retention after 1 month, with values of 3336.2 μg/cm^2^ (sepiolite), 5099.9 μg/cm^2^ (kaolinite), and 3835.6 μg/cm^2^ (vermiculite), which subsequently decreased (Figure 2a). Sulfate accumulation peaked after 12 months, with maximum values of 6863.7 μg/cm^2^ (sepiolite), 3338.3 μg/cm^2^ (kaolinite), and 3296.5 μg/cm^2^ (vermiculite).

However, the type of clay mineral exerted a noticeable impact on the ion retention properties, salt resistance, and final extraction efficiency of the main salt, halite. The sepiolite poultice exhibited the lowest performance with signs of salt-weathering after 1 month, manifested as sanding, and also showed the lowest total salt extraction amount (Figure 2a; Supplementary Figure S1). This poultice reached its maximum salt accumulation after 6 months (Supplementary Figure S1). However, the quantity of salts stored was found to be considerably lower in comparison to the other two poultice mixtures (Figure 2a), as demonstrated by the ion chromatography results, which displayed a reduced capacity for the extraction of sodium ions (max. 11008.8 μg/cm2) and chloride ions (max. 38896.8 μg/cm2) in comparison with the other two mineral clays.

The kaolinite poultice displayed a salt extraction capacity of max. 111805.9 μg/cm^2^ mass eq. NaCl and a higher resilience to salt pressure, although its storage capacity was smaller to that of the vermiculite poultice (Supplementary Figure S1). Furthermore, the results of the analyses conducted after 6 months, showed the highest salt accumulation load (Figure 2a), with the highest amount of retained sodium (29716.8 μg/cm^2^) at this time and chloride (71007.8 μg/cm^2^) after 12 months of the treatment (Supplementary Table S1).

Finally, the vermiculite poultice demonstrated the most effective extraction and storage capacity (max. 118274.1 μg/cm^2^ mass eq. NaCl), as well as the higher resistance to salt-weathering by maintaining an intact surface and structure throughout the entire study period (Supplementary Figure S1). This poultice retained a max. of sodium (27367.2 μg/cm^2^) after 6 months and a max. of chloride (76798.2 μg/cm^2^) after 12 months of treatment (Supplementary Table S1).

Regarding the effect of the poultices on the treated surfaces, a reduction in sodium concentration (from 7754.4 μg/cm^2^ to 3009.6 μg/cm^2^ for sepiolite) and stability in chloride values (from 12550.8 μg/cm^2^ to 11803.2 μg/cm^2^ for vermiculite) were observed. However, an increase in other ions, particularly potassium, was detected, with concentrations reaching up to 40 times the initial values (Supplementary Table S1).

#### 3.1.2 Charterhouse of Mauerbach

The test areas at the Charterhouse Mauerbach were located in two distinct rooms, labeled M4 and M6, which presented distinct initial salt conditions. M4 had minimal salt contamination, primarily gypsum-related ions (Ca^2+^ and SO₄^2−^), whereas M6 exhibited extensive salt efflorescence, dominated by sulfates, potassium, and sodium ions (Supplementary Table S1).

##### 3.1.2.1 Test area M4

Since M4 contained mainly gypsum-related salts, it was expected that the poultices would primarily accumulate calcium and sulfate ions (Figure 2b). Indeed, the results of the analyses demonstrated that all poultices primarily extracted sulfate, with maximum values of 763.9 μg/cm^2^ (sepiolite), 379.0 μg/cm^2^ (kaolinite), and 524.4 μg/cm^2^ (vermiculite). Calcium retention was highest in kaolinite after 6 months (444.0 μg/cm^2^). However, the accumulation of calcium ions in the poultices was found to be lower than after the treatment directly on the wall.

However, some differences were observed on the retention of other ions depending on the mineral clay used. The sepiolite poultice accumulated the highest concentrations of most ions after 1 month, except for sulfate (with a maximum at 6 months) and chloride (which peaked at 12 months, with 191.0 μg/cm^2^) (Supplementary Table S1; Figure 2b).

The kaolinite-based poultice exhibited a distinct behavior. The highest concentration of all total ions was observed after 6 months, with the exception of nitrate ions, which showed their maximum (39.3 μg/cm^2^) after 1 month (Supplementary Table S1; Figure 2b). The kaolinite poultice extracted a multiple of the salt ions originally present in the substrate, with the amount of sodium ions extracted reaching up to 170 times the original quantity. However, the amount of extracted sulfates was smaller after 12 months compared to those after 1 and 6 months (Figure 2b).

Concerning the vermiculite poultice, the highest concentrations of sodium (49.2 μg/cm^2^), potassium (109.4 μg/cm^2^), and magnesium (56.2 μg/cm^2^) ions were observed after a period of 1 month (Supplementary Table S1; Figure 2b). Sulfate and calcium showed their maximum retention at 6 months, and the highest quantities of nitrates (17.8 μg/cm^2^) and chlorides (20.6 μg/cm^2^) were recorded after 12-month period. In comparison to the initial value on the non-treated surface area (NT), the poultice demonstrated the greatest storage capacity for magnesium ions after 1 month, reaching a concentration up to 14.1 times the initial value (Supplementary Table S1).

At the end of treatment, the treated surfaces exhibited increased sulfate (1180.8–2001.6 μg/cm^2^), calcium (423.4–837.6 μg/cm^2^), and magnesium (50.4–76.8 μg/cm^2^) concentrations, regardless of the poultice used (Figure 2b). A slight increase in chloride ions was observed in the area treated with the sepiolite poultice, while the original value of potassium ions was found to be found approximately fourfold in the area treated with the vermiculite poultice (Supplementary Table S1).

##### 3.1.2.2 Test area M6

In the non-treated M6 test area, the most prevalent ions detected before treatment were sulfate (7135.2 μg/cm^2^), potassium (2918.4 μg/cm^2^), and sodium (1022.4 μg/cm^2^) with a minor presence of calcium (62.8 μg/cm^2^) (Supplementary Table S1). These ions were also identified in considerable quantities within all poultices. However, the poultices also demonstrated the accumulation of a greater quantity of nitrate, chloride, and magnesium ions than initially present in the test area (Figure 2c). At the end of the monitoring campaign (12 months) all three poultices exhibited the highest accumulation of the dominant salt ions within their matrix, specifically sulfate (1842.2–2937.6 μg/cm^2^), potassium (278.4–1452.0 μg/cm^2^), and sodium (487.2–1356.0 μg/cm^2^). The maximum concentration of chloride ions in all poultices was detected after 6 months (51.6–197.0 μg/cm^2^).

Regarding salt accumulation in the poultices, the highest quantity of extracted salts was observed in the mineral poultice made of kaolinite after a 12-month period (Figure 2c). The kaolinite-based poultice exhibited superior efficacy in accumulating the dominant species of ions (sulfate, potassium and sodium), present on the non-treated surfaces. In contrast, the sepiolite-based poultice was more effective in accumulating chloride ions. Notably, individual variations were observed among the poultices. The highest concentration of magnesium ions (85.4 μg/cm^2^) was detected in the sepiolite poultice after 1 month, whereas in the kaolinite poultice, the highest concentration (8.4 μg/cm^2^) was recorded after 6 months. Regarding nitrate extraction, the vermiculite poultice showed the highest retention after 1 month (63.7 μg/cm^2^), whereas sepiolite and kaolinite reached a maximum after 12 months (72.1 and 41.9 μg/cm^2^, respectively; Supplementary Table S1).

At the end of the treatment, compared to the non-treated (NT) areas, the treated (T) areas exhibited a significant decrease in the concentrations of weathering ions. Sulfate decreased from 7135.2 μg/cm^2^ at the NT surface to 6.2–350.4 μg/cm^2^ at the surface, sodium from 1022.4 to 8.6–205.4 μg/cm^2^, potassium from 2918.4 to 7.7–159.6 μg/cm^2^, and calcium from 62.8 to 16.8–23.5 μg/cm^2^, regardless of the poultice used (Figure 2c). Furthermore, a decrease in nitrate and chloride concentrations was observed in the treated areas with kaolinite and vermiculite, while a slight increase in magnesium ions was detected in all treated areas (Supplementary Table S1).

### 3.2 Effect of the desalination treatment on the surface-associated microbial communities: a metagenomic monitoring

The second objective of this monitoring study was to investigate how the changes in ion concentrations, during and after treatment, affected the salt-associated biofilms on the treated surfaces. For this purpose, the microbial communities present on the walls before and after treatment, as well as on the different mineral poultices after 1, 6, and 12 months of their application, were monitored using a metagenomic approach. Therefore, long-read archaeal- and bacterial-16S rRNA amplicon analysis employing the Nanopore sequencing technology was conducted, as explained in the methods section. The microbial communities present on the walls before treatment (indicated as non-treated) were previously identified and their detailed composition is shown in Tichy et al. (2023).

#### 3.2.1 Metagenomic monitoring in St. Virgil Chapel

##### 3.2.1.1 Bacterial monitoring

**Figure 3:**
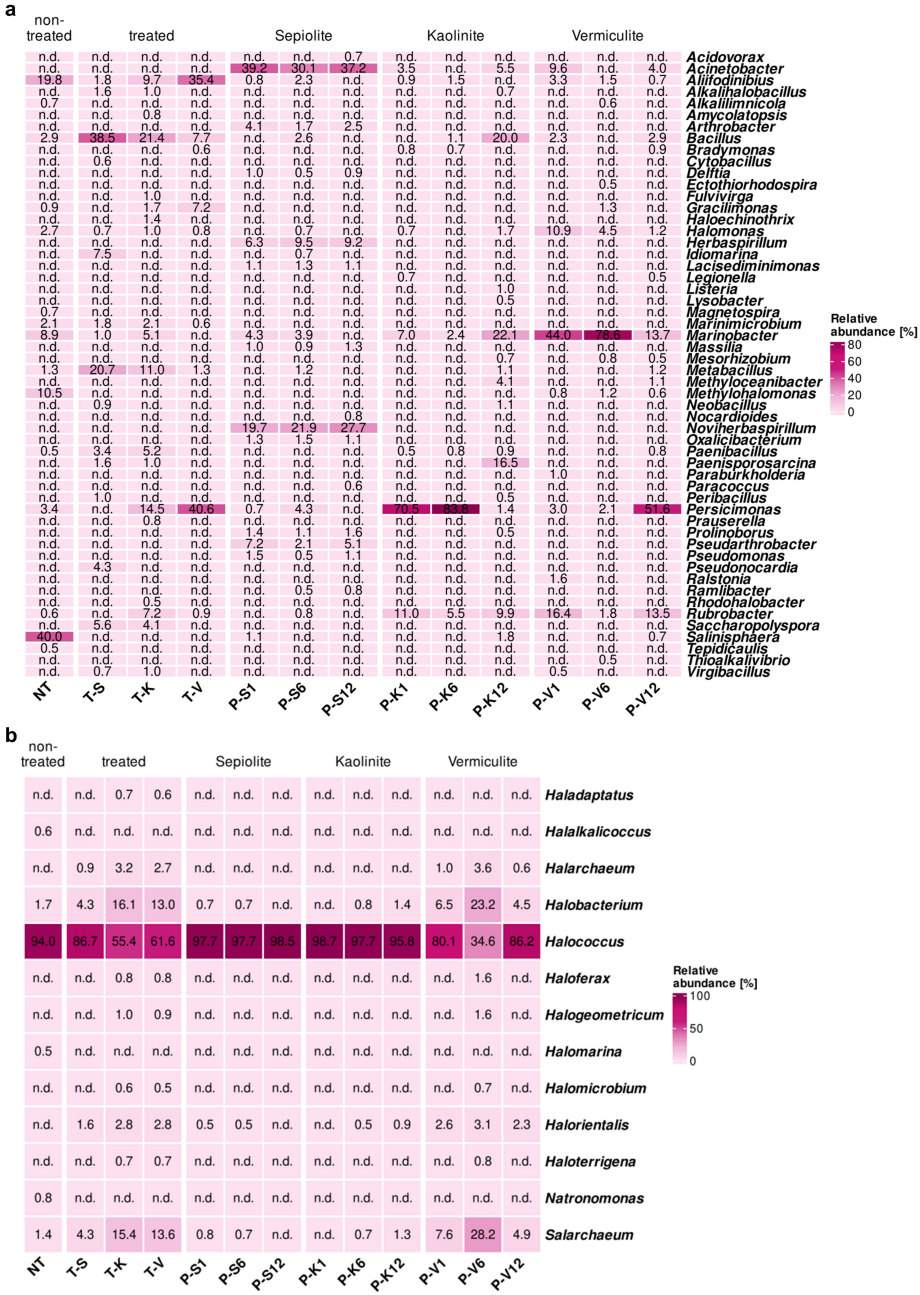
Heatmaps showing the relative abundance in percentages of **(a)** bacteria and **(b)** archaea, at the genus level (cut-off at 0.5%) for samples taken at the St. Virgil Chapel [V2]. Heatmap colors correspond to the abundance values, the darker the color, the higher the relative abundance. NA denotes measurements where the given genus was not detected above the set cut-off value. The samples include following letters for further description: Poultice [P]; sepiolite [S]; kaolinite [K]; vermiculite [V]; non-treated [NT], after the treatment [T + S/K/V] within the time intervals after 1, 6, and 12 months.

The treatment with mineral poultices showed promising results for the elimination/mitigation of some of the genera that were most abundant on the V2 surface in the Virgil Chapel before treatment. These genera, in particular, *Salinisphaera*, *Methylohalomonas*, *Marinobacter*, *Halomonas*, *Marinimicrobium*, and *Aliifodinibius*, generally showed a decrease in their relative proportions, or even in some cases, a complete elimination from the wall after treatment (Figure 3a). *Salinisphaera* and *Methylohalomonas*, which formed 40 and 10.5% of the microbial community on the wall before treatment, disappeared completely from the surfaces after treatment, irrespective of the used poultice. During treatment, *Salinisphaera* was detected in the poultices sporadically and in low proportions, i.e., in the sepiolite after 1 month (1.1%), and in the kaolinite and vermiculite after 12 months (1.8 and 0.7%, respectively). *Methylohalomonas* was only detected in the vermiculite poultice, showing low proportions over time (0.6–1.2%). *Marinobacter*, *Halomonas* and *Marinimicrobium* showed a significant decrease in their relative proportion on the treated walls. *Marinobacter* was even eliminated after treatment with vermiculite. However, the proportions of these genera within the poultices throughout the treatment was different. *Marinobacter* was detected inside all three poultices over time with relatively low concentrations in the case of sepiolite (3.9–4.3%), but an enrichment of this genus was seen within the kaolinite (7.0–22.1%) and vermiculite poultices (13.7–78.6%). The same trend was observed with the genus *Halomonas*, which also showed an enrichment within the vermiculite poultice (1.2–10.9%). In contrast, Marinimicrobium was not detected in any of the poultices (Figure 3a). The genus *Aliifodinibius* (19.8% before the treatment) showed a different behavior depending on the poultice used. After treatment with sepiolite- and kaolinite-poultices, there was a decrease in its proportion on the walls (1.8 and 9.7%, respectively), but an increase after treatment with vermiculite (up to 35.4%). Furthermore, this genus was detected inside all the poultices with proportions ranging from 0.7 to 3.3%. Finally, some other genera that showed a low abundance in the untreated surface community (below 1%), such as *Alkalilimnicola*, *Magnetospira* and *Tepidicaulis* also disappeared completely from the treated surfaces and were not detected during treatment in the poultices, with the exception of *Alkalilimnicola*, which was detected in the vermiculite poultice at 6 months of treatment in low proportion (0.6%).

In contrast, an enrichment was observed for other genera, compared to the surfaces prior treatment (Figure 3a). *Bacilli*, including members of the genera *Bacillus*, *Metabacillus*, and *Paenibacillus* showed an increase on treated surfaces, especially on areas treated with sepiolite (up to 38.5, 20.7, and 3.4%, respectively) and kaolinite (up to 21.4, 11.0, and 5.2%, respectively). During the treatment, members of *Metabacillus* and *Paenibacillus* were scarcely detected within the poultices with low relative proportions at different monitoring times, but *Bacillus* showed to be especially enriched in the kaolinite poultice after 12 months (20.0%). Members of *Gracilimonas* and *Persicimonas* showed the same trend, with an increase in their relative proportions in post-treated surfaces with kaolinite (up to 1.7 and 14.5%, respectively) and even higher with vermiculite (7.2 and 40.6%, respectively), but undetected in sepiolite-treated areas (Figure 3a). However, during the treatment these two genera showed a different trend, *Gracilimonas* was only identified after 6 months within the vermiculite poultice at a low rate (1.3%), while *Persicimonas* showed in general a strong enrichment in the mineral poultices, especially in kaolinite (70.5–83.8% in the first half of the treatment) and vermiculite after 12 months (51.6%). Finally, *Rubrobacter* increased its relative abundance on kaolinite-treated surfaces (from 0.6 to 7.2%), and showed an enrichment in the kaolinite and vermiculite poultices during the treatment (ranging from 1.8 to 16.4%).

In addition, members of 15 other genera, which were either below the detection level or not present on the untreated walls, emerged in the bacterial community of the treated walls, especially in the kaolinite- and/or sepiolite-treated surfaces with low relative proportions (0.5–7.5%). Among them, were mainly members of the *Actinomycetota* phylum, such as *Haloechinothrix*, *Pseudonocardia* and *Saccharopolyspora* in addition of *Alkalihalobacillus*, *Idiomarina* and *Paenisporosarcina*. Of these only *Paenisporosarcina* showed a significant enrichment within the vermiculite poultice reaching a proportion of 16.5% at the end of the treatment. Some genera were generally detected only sporadically and in very low proportions (≤1%), these include members of the *Bacillota* phylum, such as *Cytobacillus*, *Neobacillus*, *Peribacillus*, and *Virgibacillus* in addition to *Amycolatopsis*, *Bradymonas*, *Fulvivirga*, *Prauserella*, and *Rhodohalobacter* (Figure 3a).

Interestingly, some genera were identified exclusively within the mineral poultices, which were not detected on the wall surfaces either before or after treatment (Figure 3a). In this regard, only the genus *Acinetobacter* was detected within all three mineral clays, with the highest relative abundance in sepiolite (39.2%). However, in general, the genera detected exclusively in the mineral poultices were different according to the type of clay, indicating their possible origin in their mineral composition. The poultice with the highest number of bacterial genera was composed of sepiolite and contained the following genera: *Arthrobacter* (1.7–4.1%), *Herbaspirillum* (6.3–9.5%), *Lacisediminimonas* (1.1–1.3%), *Massilia* (1.0–1.3%), *Noviherbaspirillum* (19.7–27.7%), *Oxalicibacterium* (1.1–1.5%), *Pseudoarthrobacter* (2.1–7.2%) and *Pseudomonas* (1.1–1.5%). In addition, the genus *Prolinoborus* (1.1–1.6% within the sepiolite poultice), was also detected in the kaolinite poultice after 12 months of treatment, but with a very low proportion (0.5%). The genus *Methyloceanibacter* was detected within the poultices made of kaolinite and vermiculite (4.1 and 1.1%, respectively, after 12 months) and *Ralstonia* was found only in the vermiculite clay at the beginning of the treatment (1.6%) and was not detectable thereafter.

##### 3.2.1.2 Archaeal monitoring

The archaeal community composition of the non-treated surfaces in area V2 was clearly dominated by the genus *Halococcus* (94.0% of the total archaeal community) (Figure 3b). Interestingly, the relative abundance of this genus was decreased in the treated surfaces after the application of any mineral poultice, showing a reduction to 86.7% (sepiolite), 61.6% (vermiculite) and 55.4% (kaolinite). Monitoring of the poultices during the period of treatment showed a strong enrichment of *Halococcus* within the sepiolite and kaolinite poultices, with relative values above 95% of the population. In the vermiculite poultice, a different behavior was observed over time with values ranging from 34.6 to 86.2%. Furthermore, the genera *Natronomonas*, *Halalkalicoccus* and *Halomarina* (0.8, 0.6, and 0.5% in non-treated surfaces, respectively) were completely eliminated from the surfaces treated with the different poultices and were not detected within any of them along the treatment (Figure 3b).

In contrast, the genera *Halobacterium* and *Salarchaeum*, which showed the second highest abundance on the untreated surface (1.7 and 1.4%, respectively), underwent an increase in relative abundance on the treated walls, with values ranging from 4.3 to 16.1%, depending on the poultice used (Figure 3b). Their monitoring within the poultices showed negligible values in the sepiolite and kaolinite clays, but a significant enrichment in the vermiculite poultice.

Other genera, such as *Haladaptus*, *Halarchaeum*, *Haloferax*, *Halogeometricum*, *Halomicrobium*, *Halorientalis*, and *Haloterrigena* appeared sporadically in some of the treated areas and poultices, with relatively low proportions (below 4%), indicating a slight increase in diversity in the archaeal community during and after the treatment.

#### 3.2.2 Metagenomic monitoring in Charterhouse Mauerbach

##### 3.2.2.1 Testing location M4

**Figure 4:**
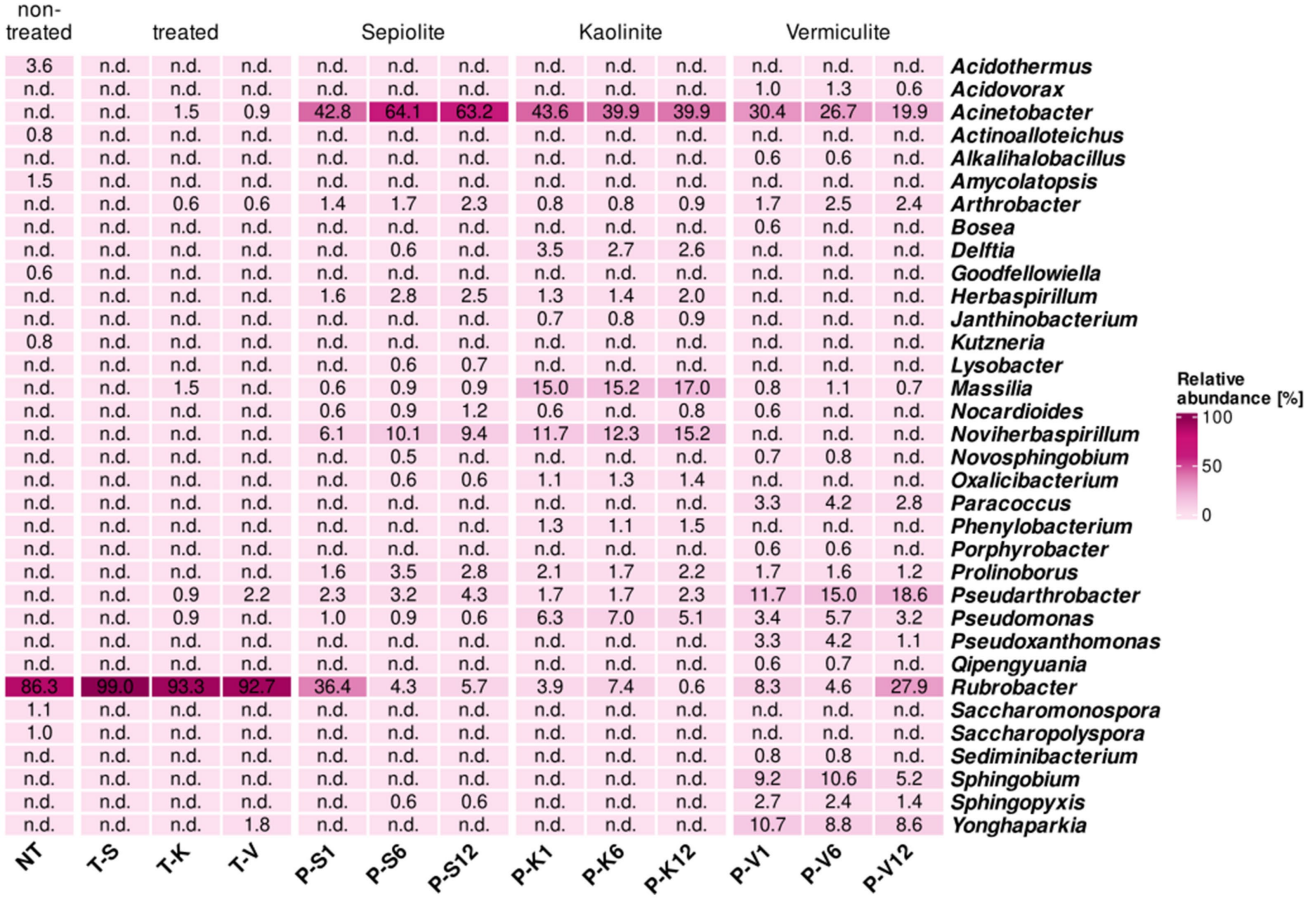
Heatmap showing the relative abundance in percentages of bacteria at the genus level (cut-off at 0.5%) for samples taken at the Charterhouse Mauerbach [M4]. Heatmap colors correspond to the abundance values, the darker the color, the higher the relative abundance. NA denotes measurements where the given genus was not detected above the set cut-off value. The samples include following letters for further description: Poultice [P]; sepiolite [S]; kaolinite [K]; vermiculite [V]; non-treated [NT], after the treatment [T + S/K/V] within the time intervals after 1, 6, and 12 months.

Seven genera that were present on the non-treated M4 test surface, with relatively low proportions (0.6–3.6%), were no longer detected on its surface during and after the desalination treatment, these genera were *Acidothermus*, *Actinoalloteichus*, *Amycolatopsis*, *Goodfellowiella*, *Kutzneria*, *Saccharomonospora*, and *Saccharopolyspora*. In contrast, *Rubrobacter*, the most dominant genus on the surface of M4 before the desalination treatment (86.3%), showed an increase in its relative abundance on the treated surfaces ranging from 92.0 to 99.0%, while within the mineral poultices, *Rubrobacter* only showed a remarkably high proportion in sepiolite after 1 month (36.4%), and in vermiculite after 12 months (27.9%) (Figure 4).

In addition, members of 6 other genera, not detectable in the non-treated walls, were found in the bacterial community of the vermiculite- and/or kaolinite-treated walls with low relative proportions (0.6–2.2%). These are *Acinetobacter*, which also showed a massive enrichment in all poultices during treatment (from 19.9 to 64.1%); *Arthrobacter*, also detected within all mineral clays (0.8–2.5%); *Massilia*, only present on the kaolinite-treated surface and enriched in this poultice (15.0–17.0%), *Pseudarthrobacter*, which showed a significant enrichment in the vermiculite poultice (11.7–18.6%); *Pseudomonas*, with a higher enrichment in the kaolinite poultice (5.1–7.0%), and *Yonghaparkia*, associated with the vermiculite poultice with percentages ranging 8.6–10.7% and transferred to the treated surface (1.8%) (Figure 4).

Similar to what was observed in the St. Virgil Chapel, some additional genera were detected only within the poultices, which were not being transferred to the walls during the treatment. These genera seem to be specifically related to the clay component of each poultice. Thus, in the sepiolite and kaolinite clays, the following genera were enriched above 1% of the population, respectively: *Delftia*, *Herbaspirillum*, and *Noviherbaspirillum*. In the vermiculite poultice, the genera enriched over 1% of the population within the clay were *Paracoccus*, *Pseudoxanthomonas*, *Sphingobium*, and *Sphingopyxis* (Figure 4).

Concerning the archaeal community at test site M4, members of this community were shown to be at the limit of PCR detection, and therefore, results were not consistent for reliable identification, neither during the desalination treatment on the poultices, nor after treatment on the treated surfaces (see also section 3.3. Quantitative PCR analyses).

##### 3.2.2.2 Testing location M6

**Figure 5:**
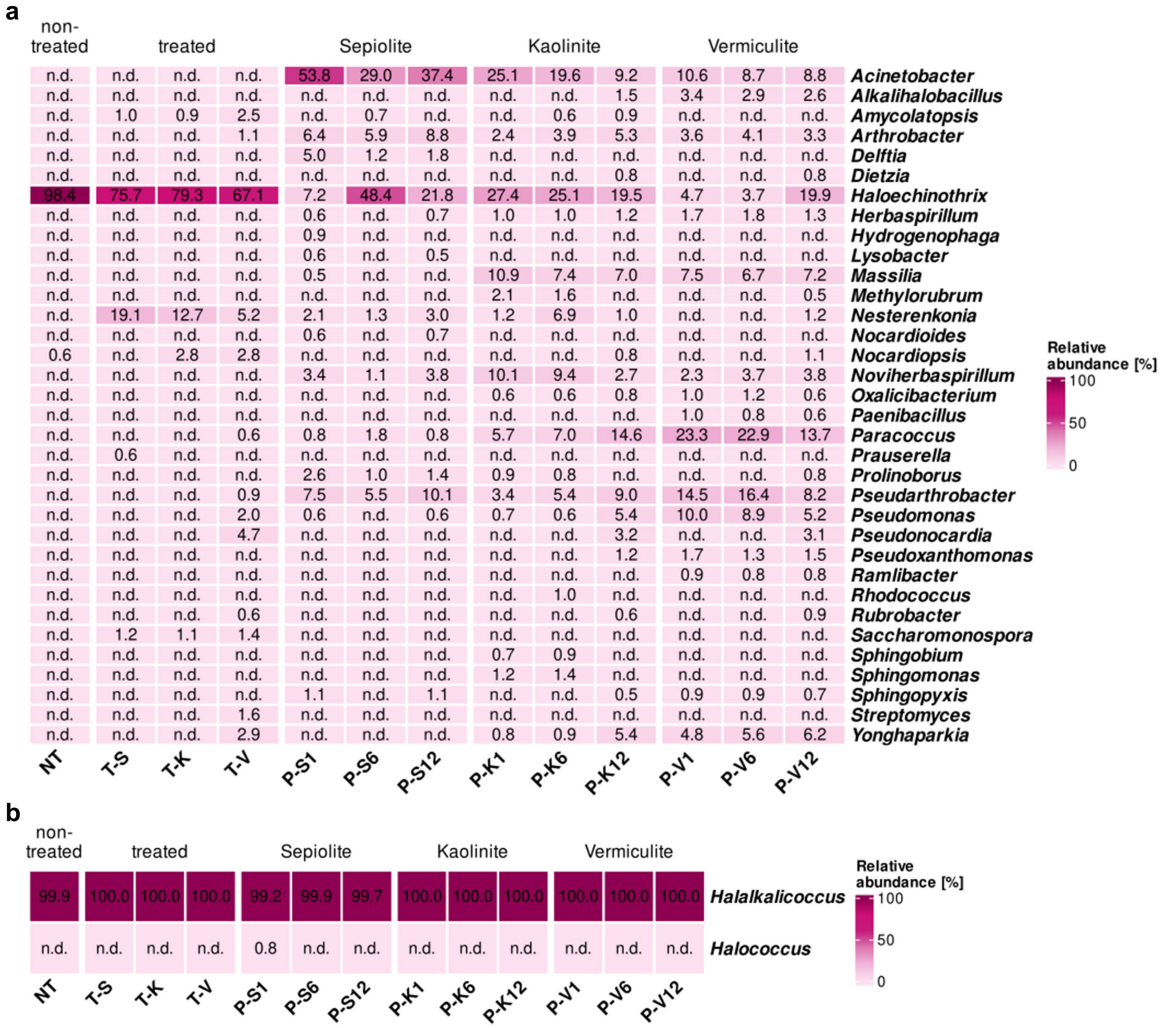
Heatmap showing the relative abundance in percentages of **(a)** bacteria and **(b)** archaea, at the genus level (cut-off at 0.5%) for samples taken at the Charterhouse Mauerbach [M6]. Heatmap colors correspond to the abundance values, the darker the color, the higher the relative abundance. NA denotes measurements where the given genus was not detected above the set cut-off value. The samples include following letters for further description: Poultice [P]; sepiolite [S]; kaolinite [K]; vermiculite [V]; non-treated [NT], after the treatment [T + S/K/V] within the time intervals after 1, 6, and 12 months.

The surface before and after the desalination treatment of the testing location M6 was dominated by one highly abundant genus, *Haloechinothrix*. However, this genus faced a reduction from 98.4% toward the range of 67.1–79.3% in the treated surfaces (Figure 5a). Moreover, *Haloechinothrix* showed a significant enrichment in the mineral poultices of sepiolite (up to 48.4% after 6 months), kaolinite (19.5–27.4%), and partially in vermiculite (4.7–19.9%). In contrast, *Nocardiopsis*, also detected on the non-treated surface (0.6%), increases in proportion (up to 2.8%) on the surfaces treated with kaolinite and vermiculite but disappears from the sepiolite-treated surface.

In addition, members of 12 other genera, which were either below the detection level or not present on the untreated walls in M6, were detected in the bacterial community on the treated walls (Figure 5a). These genera appear sporadically on one or the other treated surfaces with relative abundances below 5%, except *Nesterenkonia*, which was found on all treated walls with proportions between 5.2 and 19.1% and was also detected in all mineral poultices, especially in the one composed of kaolinite (1–6.9%). *Amycolaptosis* and *Saccharomonospora* were also identified on all treated surfaces but with proportions between 0.9–2.5% and 1.1–1.4%, respectively. The former was also detected during treatment within the sepiolite (6 months) and kaolinite poultices (6 and 12 months). *Prauserella* was only identified in the sepiolite-treated surface (0.6%), but not within any poultice. Interestingly, most of the newly detected genera were found on the vermiculite treated surface, such as *Arthrobacter* (1.1%), which was also detected within all of the mineral poultices, with similar proportions ranging from 2.4 to 8.8%. The genera *Paraccoccus* (0.6%), *Pseudoarthrobacter* (0.9%), and *Pseudomonas* (2%) also emerged in the vermiculite-treated area, and were also detected within all mineral poultices, but always with a higher proportion within the vermiculite clay, reaching significant relative proportions ranging from 5.2 to 23.3%. *Pseudonocardia*, *Rubrobacter*, and *Yonghaparkia* (4.7, 0.6. and 2.9% on the vermiculite-treated surface, respectively) were also detected within the poultices composed of kaolinite and vermiculite, the first two genera only after 12 months of treatment (with proportions ranging 0.6–3.2%) and the latter throughout the treatment, but again showing a higher proportion in the vermiculite clay (4.8–6.2%). Finally, *Streptomyces* was identified at a relative abundance of 1.6% also in the area treated with vermiculite but was not detected in any of the poultices during the desalination treatment (Figure 5a).

Similar to what was observed at the other test sites, some additional genera were only detected inside the poultices, without transferring to the walls during treatment. Six genera were detected inside all poultices, regardless of the clay component, such as *Acinetobacter*, especially enriched in the sepiolite clay (29.0–53.8%) but also with significant proportional values in the kaolinite (9.2–25.1%) and vermiculite clays (8.7–10.6%); *Herbaspirillum* (from 0.7% in sepiolite up to 1.8% in vermiculite), *Massilia* (from 0.5% in sepiolite up to 10.9% in kaolinite) and *Noviherbaspirillum* (from 1.1% in sepiolite up to 10.1% in kaolinite). In addition, the genera *Prolinoborus* and *Sphingopyxis* were also detected in all mineral clays, but in lower proportions (between 0.5 and 2.6%). Five other genera were detected in the kaolinite and vermiculite poultices in relatively low proportions (between 0.5 and 3.4%) but not in sepiolite, namely *Alkalihalobacillus*, *Dietzia*, *Methylorubrum*, *Oxalicibacterium*, and *Pseudoxanthomonas* (Figure 5a). Finally, additional genera appeared exclusively on one or the other mineral clay at different monitoring times. Thus, in the sepiolite poultice, the genera *Delftia* (1.2–5.0%) emerged in addition to *Hydrogenophaga*, *Lysobacter*, and *Nocardioides* (all three with proportions below 1.0%). In the kaolinite poultice, the genera *Sphingomonas* (1.2–1.4%) together with *Sphingobium* and *Rhodococcus* (both with proportions ≤ 1.0%) were detected and lastly in the vermiculite poultice, the genera *Paenibacillus* and *Ramilibacter* were detected exclusively, but with proportions ≤ 1.0%.

The archaeal community on the non-treated surface of test area M6 was absolutely dominated by members of the genus *Halalkalicoccus* (99.9%) and likewise persisted during and after the desalination treatment, both within the desalination poultices as well as on the treated surfaces, irrespective of the mineral clay used (99.2–100.0%) (Figure 5b).

#### 3.2.3 Diversity indices

**Figure 6:**
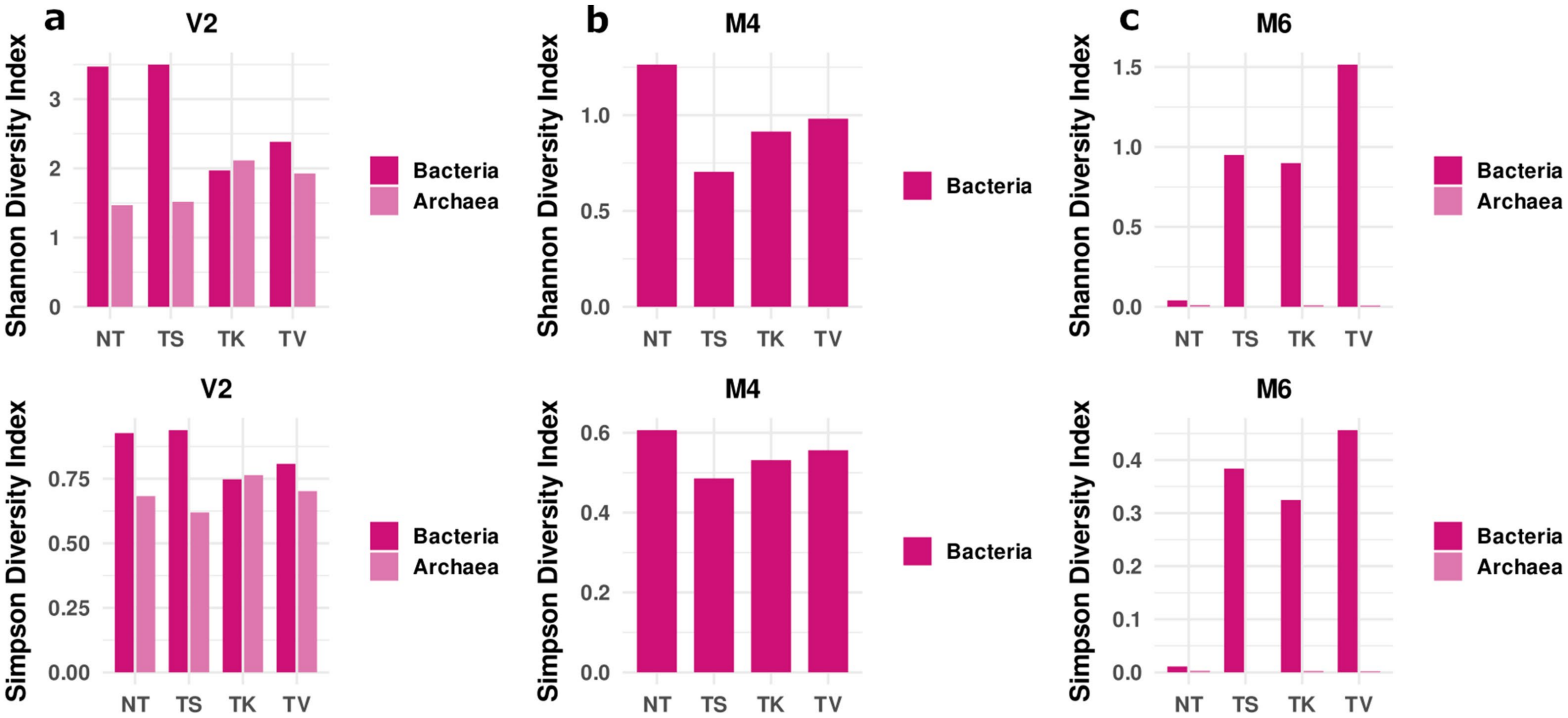
Diversity indices. The upper part shows the Shannon diversity index and the lower part shows the Simpson diversity of the sampling locations of **(a)** the St. Virgil Chapel (V2), and the Charterhouse Mauerbach: **(b)** of room M4 and **(c)** of room M6. The samples include following letters for further description: non-treated [NT]; treated [T] with: sepiolite [S]; kaolinite [K]; vermiculite [V].

In the St. Virgil Chapel, *Shannon* and *Simpson’s* diversity indices showed a higher diversity for bacteria on the non-treated surface (NT) of area V2, as well as on the surface treated with the sepiolite poultice (TS) compared to the surfaces treated with kaolinite (TK) and vermiculite (TV) (Figure 6a). In contrast, the diversity indices calculated for archaea showed a lower diversity on non-treated surfaces (NT) compared to that on treated surfaces. Only on the surface treated with sepiolite (TS), the diversity was shown to be almost equal (*Shannon’s* index) or even lower (*Simpson’s* index) after treatment (Figure 6a). The results indicate that, in general, treatment with mineral poultices led to a decrease in bacterial biodiversity and to a slightly increase in archaeal biodiversity, the effect of sepiolite not being as evident as for the other mineral clays.

In the Charterhouse Mauerbach, the diversity indices were calculated for the two rooms tested, M4 and M6. In room M4, a similar behavior to that of the V2 vault of the Virgil Chapel was observed, in terms of bacterial diversity. The highest diversity was observed on the non-treated surface (NT) compared to that of the treated surfaces. After treatment, all diversity values decreased, but interestingly here, and contrary to what was observed in V2, the greatest effect on diversity was exerted by the sepiolite poultice (TS) (Figure 6b). In this test room, diversity analyses could not be performed for archaea, as they were present close to the detection limit.

In room M6, contrary to what was observed in the other two test areas, the lowest *Shannon* and *Simpson’s* diversity indices calculated for bacteria were shown on the non-treated surface (NT), which increased significantly after treatment with all mineral clays, especially in the area treated with vermiculite (TV) (Figure 6c). The diversity of the archaeal community did not change drastically after treatment, and remained extremely low, as only one species was detected with a relative abundance above 99% during the whole monitoring period, namely *Halalkalicoccus paucihalophilus* (Supplementary Figure S5).

### 3.3 Quantitative enumeration of microbial community through qPCR analyses

qPCR analyses were performed before and after the application of the mineral poultices with samples obtained from the different tested areas of both buildings in order to have a (semi)-quantitative monitoring of the possible increase or decrease of the microbial community (bacteria and archaea) due to the treatment.

The samples for the qPCR analyses were taken from the surfaces before (non-treated: NT wall samples) and immediately after removal of the mineral poultice (treated: T wall samples). Results show that in the St. Virgil Chapel, the desalination treatment increased the bacterial Ct (cycle threshold) values on all treated surfaces, irrespective of the mineral poultice used (|1.55–4.33| ∆Ct) (Supplementary Table S2). In contrast, the results of qPCR aimed at archaeal quantification only showed a statistically relevant increase in Ct values after the treatment with kaolinite and sepiolite minerals (|1.77–2.07| ∆Ct). The general increase in the Ct values indicates a decrease in both bacterial and archaeal populations after the treatment with the mineral poultices at the St. Virgil Chapel.

In the Charterhouse Mauerbach the two tested rooms M4 and M6 showed a different performance compared toward the St. Virgil Chapel. The treated areas in room M4 showed a significant decrease of the Ct values of the bacterial populations for all types of mineral poultices, compared to the values before treatment (|1.27–2.76| ∆Ct, Supplementary Table S2). The decrease in the Ct-value indicates an enrichment of the bacterial population in M4 after treatment. In contrast, the results for the archaeal population showed steady high Ct values, with non-significant variations before and after treatment, suggesting that the population of archaea in the microbial community of this sample was close to the limit of detection.

The room M6 in the Charterhouse Mauerbach showed the opposite results compared to M4. After the desalination treatment, the bacterial Ct values in areas of M6 raised between |2.00| ∆Ct (in T area with kaolinite) and |2.36| ∆Ct (in T area with the sepiolite poultice) indicating a reduction in the bacterial population. However, in the area treated with vermiculite, no significant change was observed in the Ct values compared to those observed before treatment, indicating that this clay mineral did not cause any quantitative change in the colonizing microbial population in this area. In contrast, the Ct values of the qPCR targeting the archaeal community decreased considerably on the treated surfaces with respect to the non-treated ones, showing an average decrease of |6.5| ∆Ct in the areas treated with the sepiolite and kaolinite poultices and |5.8| ∆Ct in the area treated with the vermiculite one, indicating an increase in the archaeal population in all treated areas.

## 4 Discussion

Long-term monitoring during and after any conservation treatment is essential for assessing their effectiveness and identifying potential secondary effects on both material surfaces and associated microbial communities. Many conservation strategies primarily focus on the immediate removal of salts, often neglecting the long-term impact of ionic redistribution and microbial colonization (Verges-Belmin and Siedel, 2005; Lubelli et al., 2018). However, changes in salt composition and concentration can significantly alter the physical and chemical properties of treated surfaces, influencing their susceptibility to further deterioration (Sawdy et al., 2010). Moreover, previous studies have shown that desalination treatments can shift microbial community dynamics, which could potentially favor new biodeterioration risks (Piñar et al., 2009; Pavlović et al., 2022). Therefore, integrating long-term monitoring into preservation protocols is essential to ensure that treatments provide sustainable and effective protection and to prevent unwanted side effects. This study shows that the long-term performance of different mineral poultices varied depending on the type and initial concentration of salts present on the surfaces and the clay mineral used in the poultices.

### 4.1 Long-term monitoring at the St. Virgil Chapel

#### 4.1.1 Effectiveness of poultice treatment in test area V2

As briefly mentioned in the methods section, the chapel of St. Sant Virgil is a hypogean environment subject to frequent water ingress. This site presents a unique situation, as water infiltrates from the upper surface after heavy rainfall, carrying de-icing salts from winter applications. This process leads to inhomogeneous salt loads in the subsurface chapel and continuous formation of visible salt efflorescence. The general condition of the wall surfaces in the chapel has been previously described, as rough, highly porous and structurally weak due to progressive salt weathering (Tichy et al., 2023). Given the fragile state of the surfaces and the ongoing ingress of water and salts, a long-term treatment was chosen to use mineral poultices as a salt retention surface, aiming to mitigate the formation of salt efflorescence on the wall surfaces. Inorganic mineral poultices were selected, excluding any organic material, due to past incidents in the chapel following desalination treatments with cellulose-based wet poultices, which led to mold growth (Piñar et al., 2009). For this reason, three mineral poultices (sepiolite, kaolinite, and vermiculite) were applied for 1 year, and their salt retention capacity and resistance to salt weathering were evaluated.

The results of the qualitative and quantitative salt analyses of the three different mineral poultices on test area V2 confirmed their effectiveness in retaining the primary salt, halite (NaCl) (Figure 2a). However, the type of clay mineral significantly influenced the properties and performance of the poultices. The sepiolite-based poultice demonstrated the lowest performance on a permanently moist substrate with a highly porous substrate It exhibited signs of salt-weathering after just 1 month of treatment. Maximum salt accumulation occurred at 6 months, but the total retained salt was considerably lower compared to the other poultices. A similar trend was observed in the poultice with kaolinite content, where sodium and chloride were primarily stored during the initial 6 months, and an accumulation of sulfate became evident only after 12 months. Finally, the storage capacity of the vermiculite poultice showed to be comparable to that of the kaolinite poultice, which was previously reported for its extraction efficiency (Sawdy et al., 2010). However, the vermiculite poultice showed a steady increase in stored ions throughout the 12-month treatment period (Figure 2a), with nitrate retention peaking after just 1 month.

Regarding resistance to salt-weathering, both vermiculite- and kaolinite-base poultices showed salt efflorescences accumulating on their surfaces after 6 months However, while the vermiculite poultice maintained structural integrity, the kaolinite poultice exhibited structural damage. In summary, vermiculite showed the best performance in salt retention, likely due to its high ion-exchange capacity (Verges-Belmin and Siedel, 2005), and demonstrated the highest resistance to salt-induced degradation over the treatment period in this location.

In summary, the application of the poultices at this site proved to be successful in retaining the main weathering salt, namely halite, as well as in displacing the formation of salt efflorescence to the surface of the poultices, thus preventing its formation on the wall surfaces. However, the observed changes in the salt composition in all tested areas of V2 after treatment, regardless of the poultice used, require careful interpretation. While the reduction of sodium chloride on the wall surfaces through accumulation in the poultices is a positive outcome, the increase in magnesium, calcium and potassium ions, along with the rise in nitrate and sulfate ions can lead to new type of salt damage, the long-term effects of which remain uncertain.

These results underline the limitations of mineral poultices as a protective measure. Their effectiveness is strongly dependent on the type and concentration of salts, as well as environmental factors such as moisture ingress and substrate porosity. As shown in area V2, structural degradation of some poultice types began within a few months, limiting their long-term serviceability. Although vermiculite exhibited promising performance in both salt retention and mechanical stability, its protective capacity is temporary and contingent upon controlled environmental conditions. Consequently, in this location, the application of mineral poultices should be restricted to short- to medium-term interventions, ideally not exceeding 12 months, and should be combined with regular monitoring and timely removal. Their role as a protective system is therefore context-dependent and should not be generalized without a thorough site-specific evaluation.

#### 4.1.2 Effect of mineral poultices on the microbial community V2

As shown in the previous section, and in our previous work (Tichy et al., 2023), the St. Virgil Chapel is dominated by salt efflorescence composed mostly of halite (sodium and chloride ions, Figure 2a). Treatment with the mineral poultices mitigated the weathering damage of halite to the walls, but at the same time altered the ion composition in the walls. On the one hand, anions (nitrates and sulfates) and, on the other hand, cations (potassium, calcium and magnesium) were drastically enriched up to 40 times in the case of potassium, while chlorides showed no significant change and sodium ions were reduced up to 61.2% (Supplementary Table S1). This led to the second objective of this study, which was to monitor how the application of the poultices, and the resulting ionic changes, quantitatively and qualitatively affected the microbial community established in this hypersaline environment.

The first effect observed in terms of the size of the bacterial community, based on the qPCR results (Supplementary Figure S2) was a significant quantitative reduction after the application of all three mineral poultices. At this point it is important to mention that we are aware of the biases that can be introduced in qPCR analyses by dealing with DNA extracts from environmental samples and further amplification using 16S rDNA targeted primers. Therefore, interpretation of the results must be done carefully, considering this approach only as semi-quantitative, due to the problematic of 16S rDNA copy number variation, amplicon length and matrix effects. To similar problematics in environmental metataxonomic approaches we refer to (Whitman et al., 2018). Taking the aforementioned considerations into account, these results show that the mineral poultices were able to reduce the bacterial population size in the associated biofilm in V2.

Diversity indices indicated a reduction in the bacterial biodiversity after treatment, with the effect exerted by the sepiolite poultice being less marked (Figure 6a). The application of the poultices also affected the relative proportions of the different members of the bacterial community. The native community was characterized by four main genera 4, namely *Aliifodinibius* (*A. roseus*), *Marinobacter* (*M. salarius*), *Methylohalomonas* (*M. lacus*), and *Salinisphaera* (*S. aquimarina*), in addition to other microorganisms well adapted to hypersaline conditions, such as members of the *Halomonas*. After the desalination treatment the genera *Methylohalomonas* and *Salinisphaera* were completely eliminated from the community colonizing the walls (Figure 3a; Supplementary Figure S3A), while *Aliifodinibius roseus* was able to increase its relative abundance only on the surface treated with vermiculite but decreased its proportion in the areas desalinated with sepiolite and kaolinite. Finally, the genus *Marinobacter*, in addition to *Halomonas*, showed a decrease in their relative proportions, showing in general that all these dominant genera were affected by ion displacement on the treated walls. Even if the main salt on the walls after treatment was still halite (NaCl), in particular, the K^+^ concentration after treatment turned out to be higher (up to 40 times). As described in depth in Tichy et al. (2023), the relative composition of microbial communities in hypersaline environments can differ as a function of the concentration of individual ions, such as the presence/absence of K^+^. The increase in K^+^ ions clearly leads to a shift in the structure of the community from species that are able to tolerate high salt concentrations by favoring the synthesis or uptake of compatible solutes, such as *Halomonas* and *Marinobacter*, and hence their decrease, toward those that favor the “salt-in” strategy, i.e., that transport the available K^+^ into the cells.

On the contrary, some genera present on the non-treated surfaces with relative low proportions, such as the genera *Bacillus*, *Metabacillus*, and *Paenibacillus* (Figure 3a), in addition to others that were below the detection limit prior treatment, namely *Alkalihalobacillus*, *Cytobacillus*, *Neobacillus*, *Paenisporosarcina*, *Peribacillus*, and *Virgibacillus*, increased in their relative proportion, or emerged, in some of the treated areas, especially in the sepiolite treated one. This group of enriched microorganisms was found to belong to the phylum *Bacillota*. The same was observed for another genus belonging to the phylum *Actinomycetota*, namely *Saccharopolyspora*. All of them share the common capacity of sporulation (Yumoto et al., 2003; Heyrman et al., 2004; Heyrman, 2005; Yoon et al., 2005; Yoon and Oh, 2005; Niederberger et al., 2009; Tang et al., 2009; Shi et al., 2011; Amoozegar et al., 2013; Reddy et al., 2013; Patel and Gupta, 2020). One plausible reason why some of these genera were not detected on the non-treated surface is probably due to the fact that they were present in spore form, which increases the difficulty for DNA extraction significantly (Dineen et al., 2010). The increase in their relative abundance after the treatment may consequently be due to spore germination, following the observed ionic shift. It has been reported that spore germination is possible in hypersaline environments, as in the case within this monitoring, in which highly saline conditions persisted during and after the treatment. *In vitro* experiments have shown germination of *Bacillus* strains even with 4.8 M NaCl, due to a missing environmental salt sensing mechanism (Nagler et al., 2014). The higher relative abundances of spore-forming microorganisms, mainly at the sepiolite-treated surfaces in V2, may be due to the aforementioned ionic shift. Indeed, it has been proven that under higher saline conditions, cations play a more important role than anions in inhibiting germination (Nagler and Moeller, 2015). Moreover, it was observed that the area treated with sepiolite showed lower values of cations (sodium and potassium) than the areas treated with kaolinite and vermiculite (Figure 2a). These cations (sodium and potassium) and their homoeostasis play an important role during germination, especially in the intrinsic outflow and following energy dependent reimport of potassium (Swerdlow et al., 1981). Furthermore, during the germination process, osmoprotectants are imported into the spore, also including the de-novo synthesis of osmolytes (Nagler et al., 2016). These osmoprotectants might be provided by the native microbial community present at the tested area in V2, which contains microorganisms capable of synthesizing osmolytes, as shown in Tichy et al. (2023). However, due to the fact that the St. Virgil Chapel is a hypogean environment, osmolyte precursors may also be supplied externally, through the process of degradation of imported biogenic residues (Boch et al., 1994).

An enrichment in spore-forming bacteria, especially of the phylum *Bacillota*, might have long-term implications. In this context, the enormous biodiversity of the genus *Bacillus* comprises members that show great versatility in the production of secondary metabolites. These compounds are known for their natural antagonistic effects on other potential biodeteriogenic invaders, including fungi and bacteria (Caldeira, 2021). In addition, *Bacillus* species are known for their ability to precipitate CaCO_3_ (Jroundi et al., 2021). Due to the high salinity conditions at sampling location V2, and the fact that some carbonatogenic *Bacillus* species have been shown to be halotolerant (Jroundi et al., 2017; Saiz-Jimenez and Laiz, 2000), leads to the possible expectation, that *Bacillus* enrichment at this site does not have to be considered as a biodeterioration risk, but even as a positive effect that could contribute to the natural consolidation of the surface, thus being favorable in the restoration treatment.

A reason why the “spore-forming community” did not show to be as dominant in the areas treated with kaolinite and vermiculite might be related to the introduction of a different community dynamic through the presence of a microorganism with predatory behavior, namely *Persicimonas caeni* (Wang et al., 2020), which was already present in a moderate proportion on the V2 surface prior the treatment (3.4%) and enriched in the kaolinite- and vermiculite-treated areas, but not in the sepiolite (Figure 3a). *Persicimonas caeni* shows bacterivory, i.e., the ability to feed on prokaryotic cells, as possible feeding strategy related to the colonization of saline habitats (Mu et al., 2020). Simultaneously *Aliifodinibius roseus* doubled its relative abundance from 19.8 to 35.4% in the presence of this predator. This co-occurrence, on the level of Bradymonadaceae, was also recently reported in hypersaline soil samples (Vera-Gargallo et al., 2023). A possible explanation for this co-occurrence would be either a possible mutualistic halophilic survival strategy or that the prey–predator relationship is in favor of *Aliifodinibius*, as described elsewhere (Gong et al., 2022; Wang et al., 2024). The strong oscillations within the kaolinite and vermiculite mineral poultice, due to the presence of *Persicimonas caeni,* could be related to the “kill-the-winner” model, indicating that predatory fitness is reciprocally linked to the relative abundance of these microrganisms (Cohen et al., 2021). Since prey shape predator behavior (Chen et al., 2011), it has also been reported that intraspecific diversity might increase within communities due to predation (Nair and Velicer, 2021). However, this fact could not be reported in our study, where bacterial diversity indices of kaolinite- and vermiculite-treated surfaces in V2 presented lower numbers compared to untreated areas (Figure 6a). These results suggest that the enrichment of a predator in halophilic/halotolerant communities may present an interesting potential for mitigating certain community members and opens up future potential for shaping diversity indices within these communities on cultural heritage monument surfaces.

Finally, in the wall area treated with kaolinite, sulfate ions reached the highest concentration (Figure 2a). This coincided with a strong increase of *Rubrobacter bracarensis*, from 0.6% in the native community to 7.2% in the treated one. A possible explanation for the enrichment of the treated surface by the genus *Rubrobacter* and co-occurrence of extended amounts of sulfates will be given in detail in the section (4.2.2, sampling location M4).

Concerning the genera that were identified exclusively or mostly within the mineral poultices (Figure 3a), the most noteworthy was the presence of the genus *Acinetobacter* within all three mineral clays, especially in the sepiolite one. In the sepiolite mineral clay, *Acinetobacter lwoffii* and *johnsonii* showed together a relative abundance of more than 25% during the whole sampling campaign (Supplementary Figure S3A). A wide repertoire of functional characteristics concerning desiccation (Zeidler and Müller, 2019) and osmoregulation (Breisch et al., 2019; Zhao et al., 2023) was reported for this ubiquitous species of *Acinetobacter* (Bouvet and Grimont, 1986). Moreover, *Acinetobacter* has been reported several times in the field of cultural heritage, related to surfaces treatments (Jurado et al., 2010; Jroundi et al., 2017; Skipper et al., 2022). However, throughout the treatment, it was observed that the sepiolite poultice harbored a greater number of genera and species that were only detected specifically within its matrix, such was the case of two organisms associated with the nitrogen cycle, *Herbaspirillum seropedicae* and *Noviherbaspirillum denitrificans*. *H. seropedicae* is capable of N2 fixation (Baldani and Baldani, 1986) and shows differentiated osmoregulation strategies on the genetic level (Pedrosa et al., 2011), necessary for coping with the high salt concentrations in the mineral poultice (Supplementary Table S1). This species was also reported in other cultural heritage monuments (Haidău et al., 2022). Vice versa *N. denitrificans* shows denitrifying capabilities (Ishii et al., 2017). Other organisms present in lower proportion throughout the monitoring only within the sepiolite poultice were, *Arthrobacter*, *Lacisediminimonas*, *Massilia*, *Oxalicibacterium*, *Pseudomonas*, and *Pseudarthrobacter* (specifically *P. phenanthrenivorans*) (Busse and Schumann, 2019). Also, for that last organism, osmolyte production (Son et al., 2023) and the occurrence in the context of cultural heritage monuments was reported (Skipper et al., 2022).

The kaolinite and vermiculite matrices were mostly inhabited by three genera, namely *Marinobacter*, *Persicimomas* (*P. caeni*), and *Rubrobacter* (*R. bracarensis*) (Figure 3a; Supplementary Figure S3A). In these two poultices an interesting behavior was observed between the relative proportions of the predatory bacteria *P. caeni* and *Marinobacter* species, showing to be inversely related. Within the kaolinite mineral, a strong enrichment of the predatory bacteria was observed in the first 6 months of treatment, but their relative abundance was drastically reduced within the matrix 12 months after application (from 83.3 to 1.4%), related to a possible reduction of available prey or nutrients. This was observed in parallel with an increase in the proportion of *Marinobacter*, which is known to have a higher salt tolerance (Green et al., 2006; Shi et al., 2011; Ng et al., 2014) (Figure 3a; Supplementary Figure S3A). In contrast, in the vermiculite poultice, a strong enrichment of *Marinobacter* species was observed in the first 6 months of treatment (78.6%), along with a relatively low proportion of *P. caeni* (2.1%), which experienced a very significant increase in its proportion (51.6%) combined with a massive decrease in *Marinobacter* species (13.7%) 12 months after application. The indicative susceptibility of the genus *Marinobacter* to bacterivory was already reported elsewhere (Selivanova et al., 2024), in addition to the ability to carry predatory tools at the genetic level itself (Evans et al., 2018). This ambivalence opens an interesting topic for further research on the ability of *Marinobacter* species to act as prey or predator in halophilic environments. Furthermore, the genus *Marinobacter* has already been detected in cultural heritage hypogean environments, linked to the close sea side (Jurado et al., 2022).

In line with what was observed in the bacterial population, the archaeal community also showed a decrease in population after treatment with the mineral poultices in the St. Virgil chapel, especially in the areas treated with the sepiolite and kaolinite mineral clays, based on the qPCR results (Supplementary Figure S2; Supplementary Table S2). However, the ionic shift appeared to have a minor effect on the archaeal community structure. This community was before and after the desalination treatment highly dominated by the genus *Halococcus* (Figure 3b). This might be linked to the still favorable saline conditions, especially the high concentrations of sodium, potassium and chloride ions persisting after treatment (Supplementary Table S1). The high frequency of different *Halococcus* species within the sampling location V2 could lead to the result of an archaeal sympatric community structure, due to the assumption that these *Halococci* strains prefer similar environmental halophilic conditions. After the treatment with the sepiolite poultice, the detected *Halococcus* species were identical before/after the treatment, but with some variations in their proportions, showing a general increase in the proportion of *H. dombrowskii* and a decrease of *H. salidifoniae* and *H. gingdaonensis*, whereas the areas treated with kaolinite and vermiculite showed more evident community structure shifts (Supplementary Figure S3B). In contrast to what was observed after treatment with the aforementioned poultice, the relative proportion of *H. dombrowskii* decreased after treatment with kaolinite and slightly increased for vermiculite, and the proportions of other three species gained more importance at that area. This was the case of *Halobacterium hubeiense* with the need for NaCl in the range of 15–30% (w/v) and able to utilize many different carbon and energy sources (León et al., 2024), in addition of *Halobacterium* sp. *DL1*, which is primarily sustained by amino acids (Williams et al., 2014) and *Salarchaeum japonicum*, which similarly does not metabolize sugars (Shimane et al., 2011; Supplementary Figure S3B). Thus, the increase in protein-feeding archaeal species may be related in this treated area to the large increase in the predatory bacterium *Persicimonas caeni*, which could give rise to a new ecological niche colonized by halophilic archaea that feed preferentially on amino acids released after bacterial predation, as has also been described elsewhere (Tschitschko et al., 2016). The fact of these reported protein feeding strategies should be taken as warning, especially concerning the degradation of proteinaceous binders by microorganisms (Sterflinger and Piñar, 2013; Wu et al., 2022). These results highlight the importance of not investigating bacteria and archaea in halophilic environments individually, but simultaneously.

Concerning the genera that were mostly identified within the mineral clay matrices, these were the same genera that were dominant on the non-treated surface (*Halococcus* species), as well as those enriched throughout the treatment, such as *Halobacterium* and *Salarchaeum* in the vermiculite matrix. In addition, other genera, such as *Haladaptus*, *Halarchaeum*, *Haloferax*, *Halogeometricum*, *Halomicrobium*, *Halorientalis*, and *Haloterrigena* sporadically emerged in some of the poultice matrices, with relatively low proportions (below 4%), pointing to a slight increase in diversity in the archaeal community during and after treatment.

### 4.2 Long-term monitoring at the Charterhouse Mauerbach

#### 4.2.1 Effectiveness of desalination treatment M4

In the test area M4, the primary weathering salt contaminating the surfaces was calcium sulfate, as previously outlined. Although its concentration was low, and salt efflorescences were not evident on the surfaces, it was decided to treat them with the same mineral poultices and for the same duration to allow for comparison. This decision was also based on the known potential of sulfates to cause significant deterioration of inorganic building materials (Lubelli et al., 2018). The different clay mixtures showed varying preferences in terms of extraction and accumulation of different ions. The sepiolite-based poultice demonstrated the highest ion accumulation after 1 month, with a predominance of nitrate, calcium and magnesium ions, compared to subsequent sampling points. Sulfate ions attained their maximum level after 6 months, while chloride ions required 12 months to reach their peak (Figure 2b). The kaolinite poultice demonstrated the best overall performance, reaching its maximum salt concentration after 6 months. It preferentially accumulated potassium and sodium ions in addition to calcium and sulfate. Nitrate accumulation, as in the sepiolite poultice, was highest after 1 month (Figure 2b). The vermiculite-based poultice exhibited suboptimal outcomes, with overall salt extraction levels lower than those of the other two mixtures (Figure 2b). However, the poultice demonstrated a selective extraction of potassium and magnesium ions.

At the end of the treatment, all treated surfaces in M4 exhibited an increase in calcium and sulfate ions, as well as magnesium, compared to untreated areas (Supplementary Table S1; Figure 2b). However, the area treated with the sepiolite poultice displayed the lowest accumulation of these ions relative to the other two poultices (Figure 2b). Overall, the long-term application of mineral poultices in M4, where no initial salt efflorescence damage was present, resulted in an unfavorable outcome, with higher ion concentrations accumulating on the architectural surfaces after treatment. Consequently, the treatment did not contribute to surface consolidation and raised concerns regarding potential ionic mobilization within the construction material (Verges-Belmin and Siedel, 2005).

These observations highlight important limitations of the material. In environments without visible efflorescence or with low initial salt loads, mineral poultices may not only be ineffective but could potentially redistribute salts into the substrate, as seen in M4. This suggests that their suitability as a protective system is limited to applications where active salt transport and surface crystallization are evident.

#### 4.2.2 Effect of mineral poultices on the microbial community M4

The sampling location M4 in the Charterhouse Mauerbach exhibits a unique attribute, the very low salt concentration (mainly calcium sulfate) prior the desalination treatment and its increase after the treatment with mineral poultices, as mentioned in the section 4.2.1. Therefore, it was interesting to evaluate how the increase in this weathering salt, as well as in other ions, affects the colonizing microbial community in both qualitative and quantitative terms.

The first observation was a significative increase in the size of the bacterial community, based on the qPCR results (Supplementary Figure S2), which showed that the Ct-values decreased significantly for the treatment with sepiolite, kaolinite and vermiculite (Supplementary Figure S2; Supplementary Table S2). Regarding the effects of the increased concentration of ions on the structure of the colonizing community at this site, prior treatment, the surfaces were strictly colonized by mainly one organism of the genus *Rubrobacter*. After the application of the mineral poultices, irrespective of their mineral composition, the relative abundance of this genus was even higher (Figure 4), being correlated with the increase in sulfate, and possibly magnesium, in all treated surfaces (Figure 2b). This is also in accordance with the diversity indices calculated for this area, which showed to be lower after the treatment (Figure 6b). A plausible explanation for the correlation between the increase in sulfate and the relative proportion of *Rubrobacter* might be the reported genetic capability of members of this genus to mineralize sulfates through the use of extracellular arylsulfatases (ARS) and choline-sulfatases (Miralles et al., 2023). Furthermore, the *in vitro* growth medium for this organism has been described as needing to be supplemented with MgSO4 (Jurado et al., 2012).

The strong enrichment of *Rubrobacter* in this treated area may also be due to the fact that the mobilized sulfate ions limit the growth potential of other members of the community, even with higher water availability than chloride, as reported for *B. subtilis*, which is able to grow in simple saline brines containing more chloride ions than sulfate ions (Stevens and Cockell, 2020). The even greater presence of members of the genus *Rubrobacter* after treatment implies several negative aspects, such as increased biofilm formation and pink coloration on surfaces, two previously reported capabilities of this bacterium (Tichy et al., 2023), which could represent an increased risk of biodeterioration. Since this organism has been repeatedly detected in previous studies approaching the similar phenomenon of pinkish discoloration of cultural heritage in disparate geographic locations, (Schabereiter-Gurtner et al., 2001; Imperi et al., 2007; Laiz et al., 2009; Piñar et al., 2009; Basile et al., 2025), *Rubrobacter* is still a very interesting target for further scientific research concerning the osmoregulation and desiccation-resistance mechanisms in halophilic environments, especially in those with sulfate dominance.

Regarding the microorganisms detected within all three mineral poultices, and sporadically also on the treated walls, the most abundant genus was *Acinetobacter*, being the most frequently detected species *Acinetobacter lwoffii* and *johnsonii*. Interestingly, it was also within the sepiolite clay where the highest abundance of this genus was recorded, similar to that reported for testing site V2 (Figure 4; Supplementary Figure S4). In addition, another microorganism was detected in all three poultices, but not on the surfaces itself, *Prolinoborus fasciculus*. The detection of that species needs to be carefully taken into account, because the original publication reports no tolerance toward NaCl (Pot et al., 1992). Moreover, there was strong scientific effort and research toward its reclassification, leading to a possible re-introduction as *A. lwoffii* (Glaeser et al., 2020), but ended up as officially rejected (Arahal et al., 2023). The same applies for the genera *Arthrobacter* and *Pseudarthrobacter*, with relative abundance below 2.3% on the treated surfaces of vermiculite and kaolinite, and detected within all mineral clays in different proportions. Both genera have been detected extensively on cultural heritage monuments, (Laiz et al., 2000; Gutarowska et al., 2015; Chimienti et al., 2016; Tescari et al., 2018a; Rosado et al., 2020; Pyzik et al., 2021; Liu et al., 2023). The genus *Arthrobacter* and specifically *Arthrobacter* sp. *PGP41*, has been reported to have the capability of nitrogen fixation (Xu et al., 2018), osmolyte production based on sugars as substrate (Han et al., 2021), and the possibility of metabolizing sulfurized organic compounds (Kaur et al., 2022), which might be related to the high amount of sulfates through the whole monitoring in the sampling location M4 (Supplementary Table S1). Additional genera detected within all mineral clays were *Massilia*, with the species *Massilia agri*, which tolerates less NaCl (0–2% w/v) (Chaudhary and Kim, 2017), and *Pseudomonas*, with the species *Pseudomonas stutzeri*. This last species is well-known not only for its capability to remove nitrate salts, but also for its wide repertoire of survival mechanisms in halophilic environments, extensively reported elsewhere (Bosch-Roig et al., 2016). Furthermore, *P. stutzeri* was also used, inoculated artificially on sepiolite poultices as carrier system, for the nitrate salt removal on cultural heritage monument surfaces (Ranalli and Zanardini, 2021).

Other genera appeared sporadically in one or the other clay, such as *Delftia tsuruhatensis* (Shigematsu et al., 2003), with a reported salinity tolerance based upon NaCl up to 4% (w/v) (Li et al., 2015), and detected on porous limestone on cultural heritage monuments (Jroundi et al., 2010). Furthermore, two genera, *Herbaspirillum* and *Noviherbaspirillum*, which comprise species related to the nitrogen cycle, such as *H. seropedicae* and *N. denitrificans*, were detected within the sepiolite and kaolinite clays (Figure 4; Supplementary Figure S4). These genera were also detected in the V2 testing site, but only within the sepiolite clay.

Interestingly some genera were only detected within the vermiculite clay, two *Paracoccus* species. One of them, *P. marcusii*, is able to tolerate NaCl up to 6% (w/v) (Harker et al., 1998) and described already on inorganic cultural heritage monuments surfaces (Ettenauer et al., 2014). The second, *P.* sp. *Arc7-R13* (Hollensteiner et al., 2023), was classified as a *P. marcusii* strain, both showing different tools on a genetic level to tolerate high salt loads (Leinberger et al., 2021). Interestingly, it was also reported for *P. marcusii*, that the formation of biofilms by this species on mortar surfaces could be a non-permeable barrier for salt ions (Lv et al., 2015). To our knowledge, this is the first report of this species in the context of a desalination treatment of surfaces affected by salt-weathering in the field of cultural heritage. The fact that this species may contribute to making an impermeable barrier for ions by forming part of a biofilm, opens the unresolved question for future research as to whether microbial biofilms could protect treated surfaces against future salt-weathering.

The genera *Sphingobium*, *Sphingopyxis*, and *Yonghaparkia* were mainly detected within the vermiculite matrix. *Sphingobium yanoikuyae*, a species detected in this study, is able to tolerate 3%, (w/v) of salt (Lee et al., 2019) and it has been reported to feed on sulphonated compounds (Aylward et al., 2013). This might be linked to relatively high amounts of sulfates present (Supplementary Table S1). Members of *Sphingopyxis* have shown the potential for heterotrophic nitrification and aerobic denitrification when using ammonium, nitrate or nitrite as the sole nitrogen source and exhibit a salinity tolerance of up to 5%. Species of this genus are able to regulate osmotic pressure and survive in high salinity environments by increasing metabolic levels of sucrose and D-tagatose. All these features make the members of this genus to be considered as excellent candidates for nitrogen removal treatment in saline environments (Chen et al., 2022). Last but not least *Yonghaparkia alkaliphila* tolerates up to 7% salt (tested as NaCl (w/v)) (Xie et al., 2021), and interestingly, elevated CO_2_ levels might be necessary for growth (Ueda et al., 2008).

Considering that the architectural surface under the mineral poultice is made of lime putty (CaCO_3_), as previously reported (Tichy et al., 2023), it could be hypothesized that the application of vermiculite on a calcium carbonate surface together with atmospheric CO_2_ and water present would at first become acidic (Kaufmann and Dreybrodt, 2007), and then the pH will be buffered by the vermiculite poultice toward neutral end even slightly alkaline, as reported for deionized water with pH 5.95 (Duman and Tunç, 2008; Indrasumunar and Gresshoff, 2013). This would possibly explain the colonization of *Yonghaparkia alkaliphila* capable of handling an alkaline pH up to 9 (Yoon et al., 2006).

#### 4.2.3 Effectiveness of desalination treatment M6

As mentioned in the results section, test area M6 was characterized by a high concentration of sulfates, in addition to sodium and potassium, and with a minor presence of calcium ions. Salt efflorescences, mainly of thenardite (Na_2_SO_4_) (Tichy et al., 2023) were visible over the entire surface of this site. Studies have shown that Na_2_SO_4_ is one of the most destructive salt for sedimentary rocks and granites (Goudie et al., 1970; Kwaad, 1970). Additionally, this was investigated through the usage of saturated solutions of Na_2_SO_4_, CaSO_4_, NaCl, and NaNO_3_, including a mixture of sea salts, evaluating the resistance of sandstones and slate (Cooke, 1979; Lubelli et al., 2018).

The results of this monitoring study showed that the kaolinite-based poultice demonstrated the highest efficiency in removing the dominant SO_4_^2−^, Na^+^ and K^+^ ions, while the vermiculite-based poultice was less effective in retaining these ions (Supplementary Figure S1). The treatment in test area M6 was the most successful in this long-term study, showing that all clay poultices reached maximum salt accumulation after 12 months and achieved an almost complete reduction of weathering salts on the treated surfaces compared to untreated areas. Overall, the kaolinite-based poultice showed the best performance, followed by the vermiculite-based one. However, the possible side effect observed after treatment in M6 was a slight increase of magnesium ions on the treated surface (Supplementary Table S1). Although this could be potentially problematic and warrants further monitoring, the extent of damage associated with magnesium sulfates, as well as potential testing biases, remains under discussion (Lubelli et al., 2018).

These findings highlight both the potential and the limitations of mineral poultices as protective treatments. While the long-term application in M6 successfully reduced the concentration of damaging salts, the duration of effectiveness appears limited to the period of application, which in this study was 12 months. The performance varied notably depending on the specific clay mineral, environmental conditions, and salt composition.

#### 4.2.4 Effect of mineral poultices on the microbial community M6

As mentioned in the previous section 4.2.3, the application of mineral poultices at test site M6 of the Charterhouse Mauerbach showed very promising results in terms of mitigation or even total removal of sulfates. Therefore, it was also interesting to know whether this treatment could also mitigate the biofilm associated with the efflorescence and how the ions shift would affect the community structure colonizing the treated surfaces.

The semi-quantitative qPCR analysis showed that the bacterial community did indeed undergo a reduction in size after 12 months of treatment on the surfaces treated with sepiolite and kaolinite, but no significant reduction on the surface treated with vermiculite (Supplementary Table S2). However, the diversity indices indicated a significant increase in bacterial biodiversity after treatment, irrespective of the mineral clay used (Figure 6c). Concerning the bacterial community structure, prior the desalination treatment the sampling location M6 was strongly dominated by one bacterial genus, namely *Haloechinothrix* (with over 98% relative proportion) (Figure 5a). Interestingly, after the treatment, the abundance of *Haloechinothrix*, with the single detected species *H. alba* (Supplementary Figure S5A) decreased to levels between 67.1 and 79.3%, depending on the mineral clay applied (see Figure 5a). Conversely, the number of bacterial community members and their associated relative abundance including the genus *Nesterenkonia*, (mainly *N. xinjiangensis*), as well as of the genus *Saccharomonospora* (*S. halophila*) and *Amycolaptosis* (*A. albispora* and *A. xuchangensis*), the last two being under the detection limit in the untreated walls, increased in all treated areas (Figure 5a; Supplementary Figure S5). According to their original description *Haloechinothrix alba* tolerates 9–23% (w/v) salt (tested with NaCl) and a pH range of 4.0–8.0 (Tang et al., 2008), whereas *Nesterenkonia xinjiangensis* present a saline tolerance of 0–25% (w/v) but a tolerance to pH 7.0–12.0 (Li et al., 2004). In addition, the other emerging species such as *S. halophila*, which was isolated from soil, tolerate 10–30% NaCl and a pH range of pH 6.0–11.0 (Al-Zarban et al., 2002) and *A. albispora*, originally isolated from deep-sea sediments, has been reported to tolerate 0–8% (w/v) salinity and pH range of 5.0–11.0 (Zhang et al., 2016). The results show that all emerging species tolerated similar salt levels to the dominant *Haloechinothrix* species, but significantly higher pH thresholds, indicating an adaptation to a more alkaline environment. As mentioned before for the M4 site, through the action of the mineral poultices on a calcium carbonate surface could raise the pH in the poultice and also transiently on the treated surfaces.

Similar to what was observed in site M4, the main genera found within some or all mineral poultices, and sporadically in low proportions in the treated surfaces were, *Acinetobacter*, *Arthrobacter*, *Herbaspirillum*, *Massilia*, *Noviherbaspirillum*, *Paracoccus*, *Pseudathrobacter*, and *Pseudomonas*, besides the dominant genus *Haloechinothrix* (Figure 5a). The characteristics of these bacteria found predominantly within the mineral clays have already been explained in the paragraph 4.2.2. Consequently, it is observed that the poultices with different mixtures of sepiolite, kaolinite or vermiculite tend to contribute to and enrich a domestic microbial community specific to each clay mineral, considering that the desalination poultices were not sterilized before use, and therefore carried their own microbial community *in situ*.

Regarding the archaeal community, the ion shift produced a significant increase in this population in all treated areas (Supplementary Figure S2), denoted by the marked decrease in Ct values obtained by qPCR analysis after treatment. However, neither the biodiversity (Figure 6c) nor the structure of this community was significantly affected. The archaeal community was shown to be dominated before, during (within the poultices), and after the desalination treatment by a single species, *Halalkalicoccus paucihalophilus*, which is able to survive between 5 and 35% (w/v) NaCl and a pH range of 6.0–8.5 (Liu et al., 2013).

Although the soluble amount of sulfate was successfully removed during treatment with the mineral poultices, there was an additional source of sulfate on the non-treated M6 surfaces, namely syngenite, which are almost insoluble in water [solubility product of −7.2] (Lothenbach and Winnefeld, 2006) (Supplementary Table S3). The formation and occurrence of syngenite on calcareous surfaces of cultural heritage has already been described (Matović et al., 2012). Taking the possible need of *Halalkalicoccus paucihalophilus* for sulfates into account (according to the isolation region of this organism rich in sulfates; Salt Lake Lop Nur; Liu et al., 2013; Xiyu, 1987), syngenite can function as hidden depot for that required mineral becoming available through biomineralization, but more scientific effort is needed to demonstrate this. In general, the shift after treatment toward microbial community members with higher pH-tolerant capacities in combination with a relatively high amount of free Ca^2+^ ions (16.8–23.5 μg/cm^2^) indicates a possible increase in alkalinity. In addition, calcium ions have a positive effect on archaeal cell aggregation (Kawakami et al., 2007). To conclude, the sulfate load was removed from the walls and the microbial communities of the biofilm were partially exchanged (bacteria against archaea), resulting in a community possibly more flexibly adapted to high alkalinity and salt fluctuations than the original community.

## 5 Conclusion

This study shows one of the few existing examples of long-term monitoring of a desalination treatment, evaluating not only salt retention capacities but also the effects of the ionic changes on microbial communities colonizing the treated surfaces. Our results show a clear influence of the type and initial concentration of weathering salts on the performance of the clay mineral used. The three monitored locations exhibited distinct scenarios, highlighting the importance of comprehensive treatment approaches.

In V2, originally dominated by halite, the treatment outcome was gradually positive. The NaCl concentration remained stable after treatment, and its weathering effect on the surfaces was mitigated by its accumulation within and on the poultices. However, an increase in other ions altered the microbial community composition. qPCR analysis showed a reduction in both bacterial and archaeal biofilms Ct values, while metataxonomic data indicated a partial decline in bacterial biodiversity, particularly in NaCl-adapted genera, alongside an increase in halotolerant and/or spore-forming microorganisms that thrived with the rise in K^+^, Ca^2+^, and Mg^2+^ or the decrease in Na^+^. Among the tested poultices, the vermiculite-based one exhibited the best salt retention capacity and the highest resistance to salt weathering over the treatment period.

In M4, the treatment yielded the least favorable outcome. In this area, which initially had a low concentration of CaSO₄, the poultices facilitated the mobilization and accumulation of salts on the wall surfaces, leading to increased concentrations of SO₄^2−^, K^+^, Ca^2+^, and Mg^2+^ after treatment. This promoted further enrichment of the dominant genus *Rubrobacter*, resulting in reduced microbial biodiversity in the biofilm. The kaolinite-based poultice demonstrated the best performance in terms of salt retention, whereas the sepiolite-based poultice caused the least accumulation of ions on the treated surface.

In M6, the treatment was the most successful. A significant reduction in SO₄^2−^, K^+^, and Na^+^ on the treated surfaces led to an increase in microbial biodiversity. The newly emerging microbial members were possibly better adapted toward a more alkaline environment, likely due to Ca^2+^ solubilization. Overall, the treatment decreased bacterial community size while increasing archaeal populations, which were more suited to the altered conditions. In this case, the kaolinite-based poultice showed the best performance in terms of salt reduction capabilities.

In summary, our findings indicate that no desalination treatment should be applied without prior assessment of the surface properties, salt composition, and colonizing microbial community. Several factors must be considered before selecting a clay material, including the physical surface conditions and the concentration and chemical composition of the salts present. Furthermore, microbial monitoring should accompany these treatments to assess how changes in the saline environment affect microbial communities, potentially favoring specific microbial groups within biofilms. Additionally, this study underscores the importance of conducting preliminary tests on localized areas before implementing large-scale treatments on historic surfaces. Finally, it is essential to report unsatisfactory results, as these contribute to informed decision-making regarding the use of specific clay materials in future conservation efforts based on empirical data.

## Data Availability

The datasets presented in this study can be found in online repositories. The names of the repository/repositories and accession number(s) can be found at: https://www.ncbi.nlm.nih.gov/, PRJNA1242577.
