# Supplementary material for: Microbial community shifts during salt mitigation treatments of historic buildings using mineral poultices: a long-term monitoring of salt and associated biofilms
Source: Front Microbiol. 2025 May 22;16:1603289. doi: 10.3389/fmicb.2025.1603289 (PMC12137350; doi:10.3389/fmicb.2025.1603289)
Supplement: Supplementary file 1 [file Data_Sheet_1.docx]

Supplementary Material

**Microbial community shifts during salt mitigation treatments of historic buildings using mineral poultices: a long-term monitoring of salt and halophilic pink biofilms.**

Johannes Tichy^1^, Beate Sipek^2^, Martin Ortbauer^2^, Lukas Fürnwein^3^, Monika Waldherr^3^, Alexandra Graf^3^, Katja Sterflinger^1^, Guadalupe Piñar^1^

^1^ Institute for Natural Sciences and Technology in the Art, Academy of Fine Arts Vienna. Schillerplatz 3, A-1010 Vienna, Austria

^2^ Institute for Conservation - Restoration, Academy of Fine Arts Vienna. Schillerplatz 3, A-1010 Vienna, Austria

^3^ Department of Applied Life Sciences/Bioengineering/Bioinformatics, FH Campus Wien, Favoritenstrasse 226, A-1100 Vienna, Austria

# Supplementary Figures and Tables

**Legend of Figures:**

**Figure S1.** Empirical evaluated salt retention capacity of mineral poultices based on mass eq. NaCl [µg/cm²] for samples taken at St. Virgil Chapel (V2) and the Charterhouse Mauerbach (M-4) & (M-6), with the mineral poultices [P] type Sepiolite (S); Kaolinite (K); Vermiculite (V) after the application time of 1; 6 and 12 months [1;6;12].

**Figure S2.** qPCR results of archaeal [Arc] and bacterial [Bac] samples originating from the locations of the St. Virgil Chapel [V2] and the Charterhouse Mauerbach [M4 & M6], showing significant differences between non-trated and treated surface samples [Sepiolite, Kaolinite and Vermiculite], indicated by the black clamps on the top with the calculated p-value. Ct values of the qPCR samples are denoted in form of a boxplot

**Figure S3.** Heatmap showing the relative abundance in % of (a) bacteria and (b) archaea on species level (cut-off at 0.1%) for samples taken at the St. Virgil Chapel (V-2) during the application of the poultices (Poultivce [P]; sepiolite [S]; kaolinite [K]; vermiculite [V]; non-treated [NT], after the treatment [T+S/K/V]) within the time intervals after one month [1m]; six months [6m] and 12 months [12m]. Colours correspond to the abundance values, the darker the colour, the higher the relative abundance. NA denotes measurements where the given species was not detected above the set cut-off value.

**Figure S4.** Heatmap showing the relative abundance in % of bacteria on species level (cut-off at 0.1%) for samples taken at the Charterhouse Mauerbach (M-4) during the application of the poultices (Poultice [P]; sepiolite [S]; kaolinite [K]; vermiculite [V]; non-treated [NT], after the treatment [T+S/K/V]) within the time intervals after one month [1m]; six months [6m] and 12 months [12m]. Colours correspond to the abundance values, the darker the colour, the higher the relative abundance. NA denotes measurements where the given species was not detected above the set cut-off value.

**Figure S5.** Heatmap showing the relative abundance in % of (a) bacteria and (b) archaea on species level (cut-off at 0.1%) for samples taken at the Charterhouse Mauerbach (M-6) during the application of the poultices (Poultice [P]; sepiolite [S]; kaolinite [K]; vermiculite [V]; non-treated [NT], after the treatment [T+S/K/V]) within the time intervals after one month [1m]; six months [6m] and 12 months [12m]. Colours correspond to the abundance values, the darker the colour, the higher the relative abundance. NA denotes measurements where the given species was not detected above the set cut-off value

## Supplementary Tables

Table S1. Quantitative salt analyses of the 3 sampling locations [V2: St. Virgil Chapel; M4 and M6: Charterhouse Mauerbach] performed by ion chromatography (IC) and continuous flow analysis (CFA) [µg/cm²]

| Location V2 | No3^-^ [µg/cm²] | Cl^-^ [µg/cm²] | So4^2-^ [µg/cm²] | Na^+^ [µg/cm²] | K^+^ [µg/cm²] | Ca^2+^ [µg/cm²] | Mg^2+^ [µg/cm²] |
| --- | --- | --- | --- | --- | --- | --- | --- |
| NT | 22.9 | 12550.8 | 577.2 | 7754.4 | 65.9 | 312.5 | 82.0 |
| P-S1 | 3336.2 | 36019.8 | 418.8 | 11008.8 | 4994.4 | 2040.0 | 4245.6 |
| P-S6 | 1196.6 | 38896.8 | 123.1 | 10660.8 | 5157.6 | 3662.4 | 5275.2 |
| P-S12 | 373.7 | 30097.6 | 6863.7 | 9648.0 | 2112.0 | 2376.0 | 1632.0 |
| P-K1 | 5099.9 | 64283.6 | 613.2 | 20361.6 | 6883.2 | 2932.8 | 6482.4 |
| P-K6 | 1081.0 | 63676.8 | 208.3 | 29716.8 | 7948.8 | 3040.8 | 4394.4 |
| P-K12 | 638.6 | 71007.8 | 3338.3 | 24792.0 | 4135.2 | 2688.0 | 2856.0 |
| P-V1 | 3835.6 | 53600.7 | 2988.8 | 19257.6 | 6902.4 | 724.8 | 3336.0 |
| P-V6 | 961.4 | 61855.2 | 277.4 | 27367.2 | 8040.0 | 1672.8 | 3664.8 |
| P-V12 | 914.4 | 76798.2 | 3296.5 | 21864.0 | 5496.0 | 3720.0 | 6360.0 |
| T-S | 427.2 | 12979.2 | 3480.0 | 3009.6 | 2169.6 | 3309.6 | 1972.8 |
| T-K | 340.3 | 13656.0 | 5292.0 | 6883.2 | 3002.4 | 2796.0 | 1358.4 |
| T-V | 419.3 | 11803.2 | 3276.0 | 5978.4 | 2856.0 | 2464.8 | 1960.8 |
|  |  |  |  |  |  |  |  |
| Location [M4] | No3^-^ [µg/cm²] | Cl^-^ [µg/cm²] | So4^2-^ [µg/cm²] | Na^+^ [µg/cm²] | K^+^ [µg/cm²] | Ca^2+^ [µg/cm²] | Mg^2+^ [µg/cm²] |
| NT | 4.9 | 4.7 | 204.5 | 6.7 | 9.1 | 80.6 | 4.0 |
| P-S1 | 187.0 | 33.6 | 670.1 | 51.6 | 60.7 | 100.6 | 132.7 |
| P-S6 | 27.5 | 30.7 | 763.9 | 8.9 | 6.0 | 0.0 | 72.7 |
| P-S12 | 111.9 | 191.0 | 103.0 | 35.8 | 13.9 | 26.2 | 123.6 |
| P-K1 | 39.3 | 29.8 | 277.4 | 38.2 | 27.1 | 90.0 | 0.0 |
| P-K6 | 4.4 | 39.4 | 379.0 | 1142.4 | 1120.8 | 444.0 | 52.1 |
| P-K12 | 27.8 | 37.9 | 55.4 | 22.8 | 24.0 | 34.1 | 12.2 |
| P-V1 | 12.7 | 13.4 | 365.8 | 49.2 | 109.4 | 33.4 | 56.2 |
| P-V6 | 2.7 | 17.3 | 524.4 | 19.2 | 36.5 | 45.1 | 21.6 |
| P-V12 | 17.8 | 20.6 | 20.6 | 39.8 | 36.5 | 7.4 | 16.6 |
| T-S | 7.0 | 12.2 | 1180.8 | 7.9 | 8.2 | 423.4 | 50.4 |
| T-K | 7.1 | 4.3 | 2001.6 | 10.1 | 12.5 | 837.6 | 66.0 |
| T-V | 1.4 | 3.6 | 1740.0 | 7.7 | 38.9 | 475.0 | 76.8 |
|  |  |  |  |  |  |  |  |
| ­Location [M6] | No3^-^ [µg/cm²] | Cl^-^ [µg/cm²] | So4^-^ [µg/cm²] | Na^+^ [µg/cm²] | K^+^ [µg/cm²] | Ca^2+^ [µg/cm²] | Mg^2+^ [µg/cm²] |
| NT­­ | 1.9 | 4.0 | 7135.2 | 1022.4 | 2918.4 | 62.8 | 4.1 |
| P-S1 | 21.2 | 22.8 | 933.6 | 411.1 | 418.1 | 8.4 | 85.4 |
| P-S6 | 16.9 | 197.0 | 1095.4 | 99.8 | 97.0 | 0.0 | 0.0 |
| P-S12 | 72.1 | 76.1 | 2460.0 | 1332.0 | 1034.4 | 32.4 | 60.0 |
| P-K1 | 17.0 | 17.3 | 2592.0 | 1112.4 | 1356.5 | 0.0 | 0.0 |
| P-K6 | 17.5 | 92.6 | 983.8 | 113.3 | 76.1 | 1.9 | 8.4 |
| P-K12 | 41.9 | 27.6 | 2937.6 | 1356.0 | 1452.0 | 0.0 | 0.0 |
| P-V1 | 63.7 | 21.6 | 717.8 | 322.3 | 154.8 | 0.0 | 29.5 |
| P-V6 | 5.2 | 51.6 | 649.2 | 81.1 | 39.1 | 0.5 | 3.1 |
| P-V12 | 58.2 | 48.2 | 1842.2 | 487.2 | 278.4 | 15.6 | 33.8 |
| T-S | 4.1 | 15.4 | 350.4 | 205.4 | 159.6 | 21.1 | 17.3 |
| T-K | 0.6 | 1.2 | 6.2 | 8.6 | 7.7 | 23.5 | 8.2 |
| T-V | 1.2 | 1.4 | 6.2 | 12.5 | 20.9 | 16.8 | 15.6 |

Table S2. qPCR results of archaeal [Arc] and bacterial [Bac] samples originating from the locations of the St. Virgil Chapel [V2] and the Charterhouse Mauerbach [M4 and M6], showing Ct values of the qPCR between non-treated and treated samples [sepiolite, kaolinite and vermiculite]

Table S3. Qualitative mineral composition performed by X-ray diffraction at the Charterhouse Mauerbach (M6) on the non-treated samples prior treatment (salt-efflorescence & mortar), identical measuring parameters as described in (Tichy et al., 2023)

| **XRD Analysis Location [M6]** | **Salt-efflorescence** | **Mortar composition** |
| --- | --- | --- |
| Halite (NaCl) | - | - |
| Sylvinite (Na-Sylvine) | - | - |
| Sylvine (KCl) | - | - |
| Saltpetre (KNO_3_) | - | - |
| Calcium nitrate Ca(NO_3_)_2_ 4H_2_O | N.I | - |
| Gypsum CaSO_4_·2(H_2_O) | - | - |
| Thenardite (Na_2_SO_4_) | +++ | - |
| Aphthitalite K_3_Na(SO_4_)_2_ | + | - |
| Syngenite (K_2_Ca(SO_4_)_2_  H_2_O) | + | - |
| Calcite (CaCO_3_) | - | + |
| Dolomite | - | + |
| Mica | - | + |
| Quartz (SiO_2_) | - | +++ |
| Alkali-feldspar | - | + |
| Plagioclase-Feldspar | - | + |

High abundance: +++; intermediate abundance: ++; low abundance: +; no interpretation possible (at the border of the detection limit): N.I; non-detected: -

Grey background: weathering salts / white background: mortar component.

## Supplementary Figures


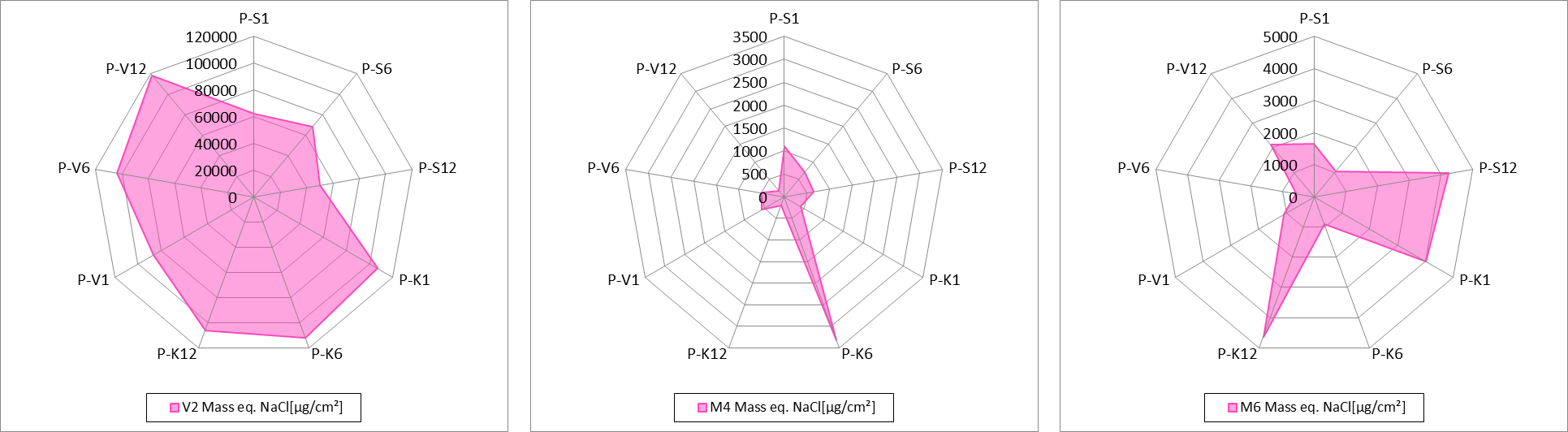


**Figure S1.** Empirical evaluated salt retention capacity of mineral poultices based on mass eq. NaCl [µg/cm²] for samples taken at St. Virgil Chapel (V2) and the Charterhouse Mauerbach (M-4) & (M-6), with the mineral poultices [P] type Sepiolite (S); Kaolinite (K); Vermiculite (V) after the application time of 1; 6 and 12 months [1;6;12].


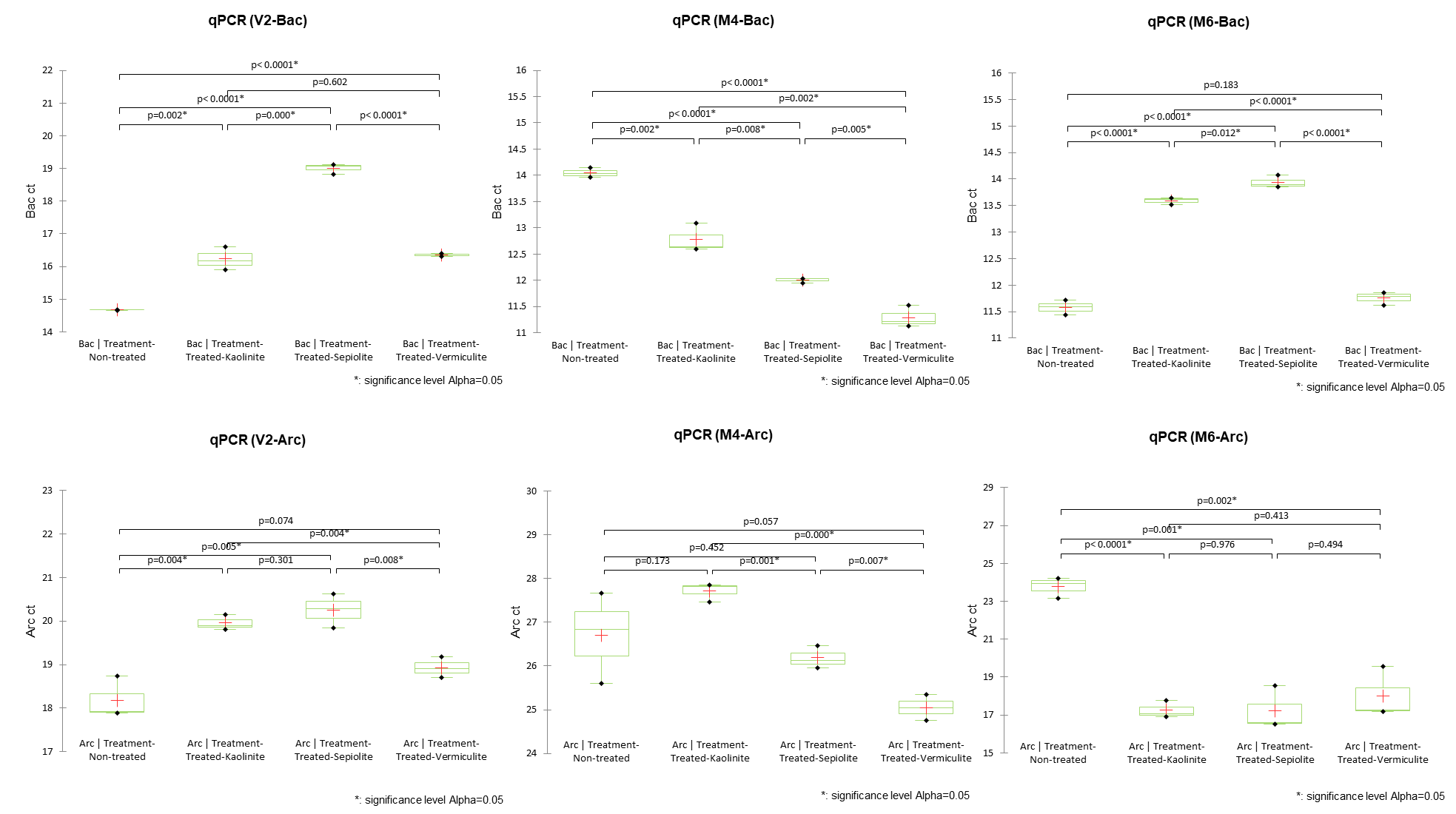


**Figure S2.** qPCR results of archaeal [Arc] and bacterial [Bac] samples originating from the locations of the St. Virgil Chapel [V2] and the Charterhouse Mauerbach [M4 & M6], showing significant differences between non-trated and treated surface samples [Sepiolite, Kaolinite and Vermiculite], indicated by the black clamps on the top with the calculated p-value. Ct values of the qPCR samples are denoted in form of a boxplot


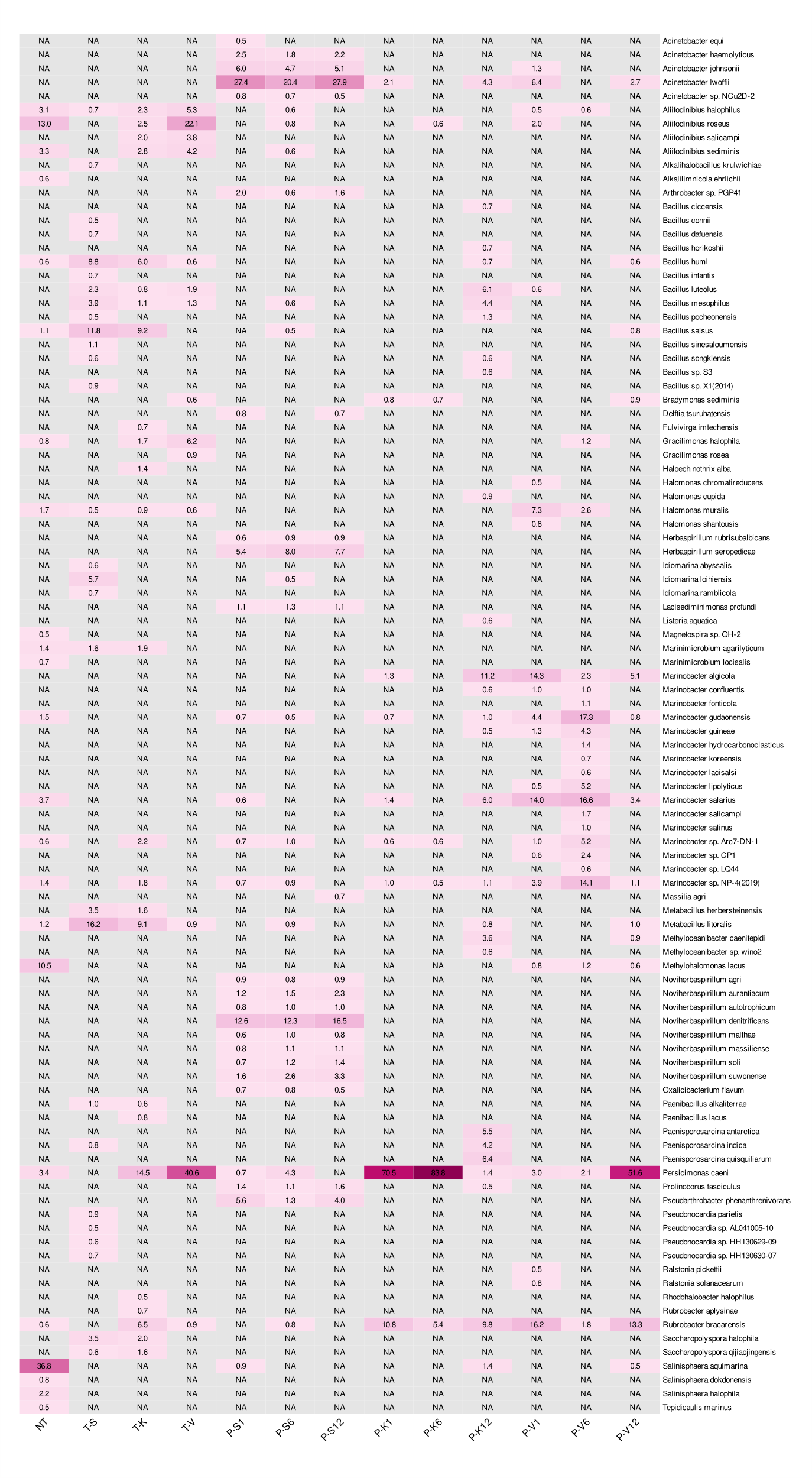


**a**

**Figure S3.** Heatmap showing the relative abundance in % of (a) bacteria and (b) archaea on species level (cut-off at 0.1%) for samples taken at the St. Virgil Chapel (V-2) during the application of the poultices (Poultivce [P]; sepiolite [S]; kaolinite [K]; vermiculite [V]; non-treated [NT], after the treatment [T+S/K/V]) within the time intervals after one month [1m]; six months [6m] and 12 months [12m]. Colours correspond to the abundance values, the darker the colour, the higher the relative abundance. NA denotes measurements where the given species was not detected above the set cut-off value.


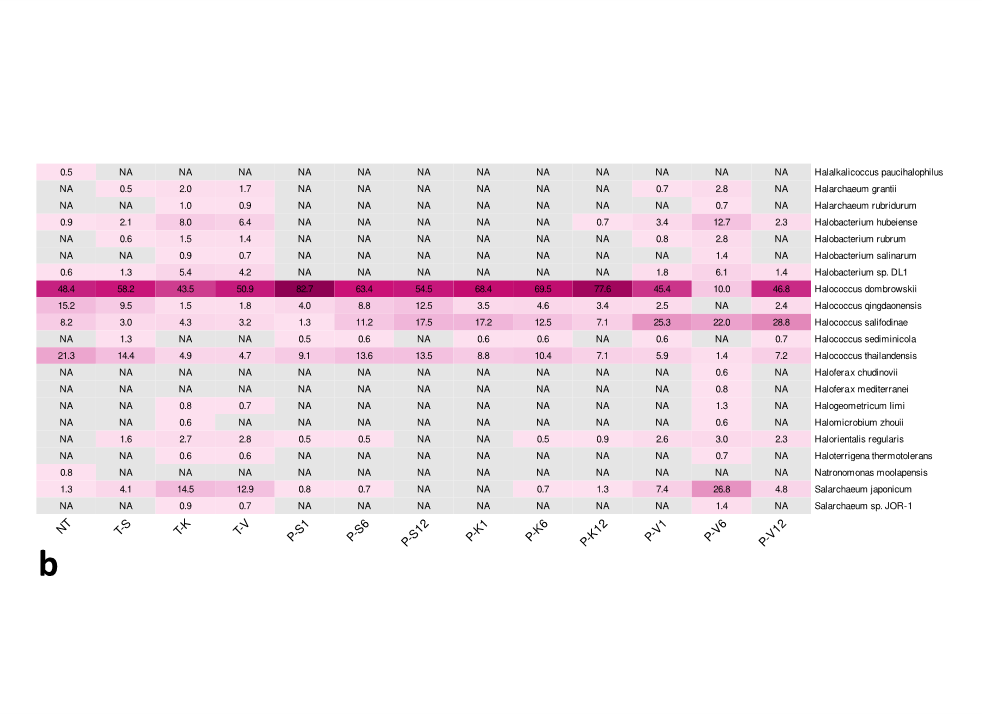
**Figure S3.** Heatmap showing the relative abundance in % of (a) bacteria and (b) archaea on species level (cut-off at 0.1%) for samples taken at the St. Virgil Chapel (V-2) during the application of the poultices (Poultivce [P]; sepiolite [S]; kaolinite [K]; vermiculite [V]; non-treated [NT], after the treatment [T+S/K/V]) within the time intervals after one month [1m]; six months [6m] and 12 months [12m]. Colours correspond to the abundance values, the darker the colour, the higher the relative abundance. NA denotes measurements where the given species was not detected above the set cut-off value.


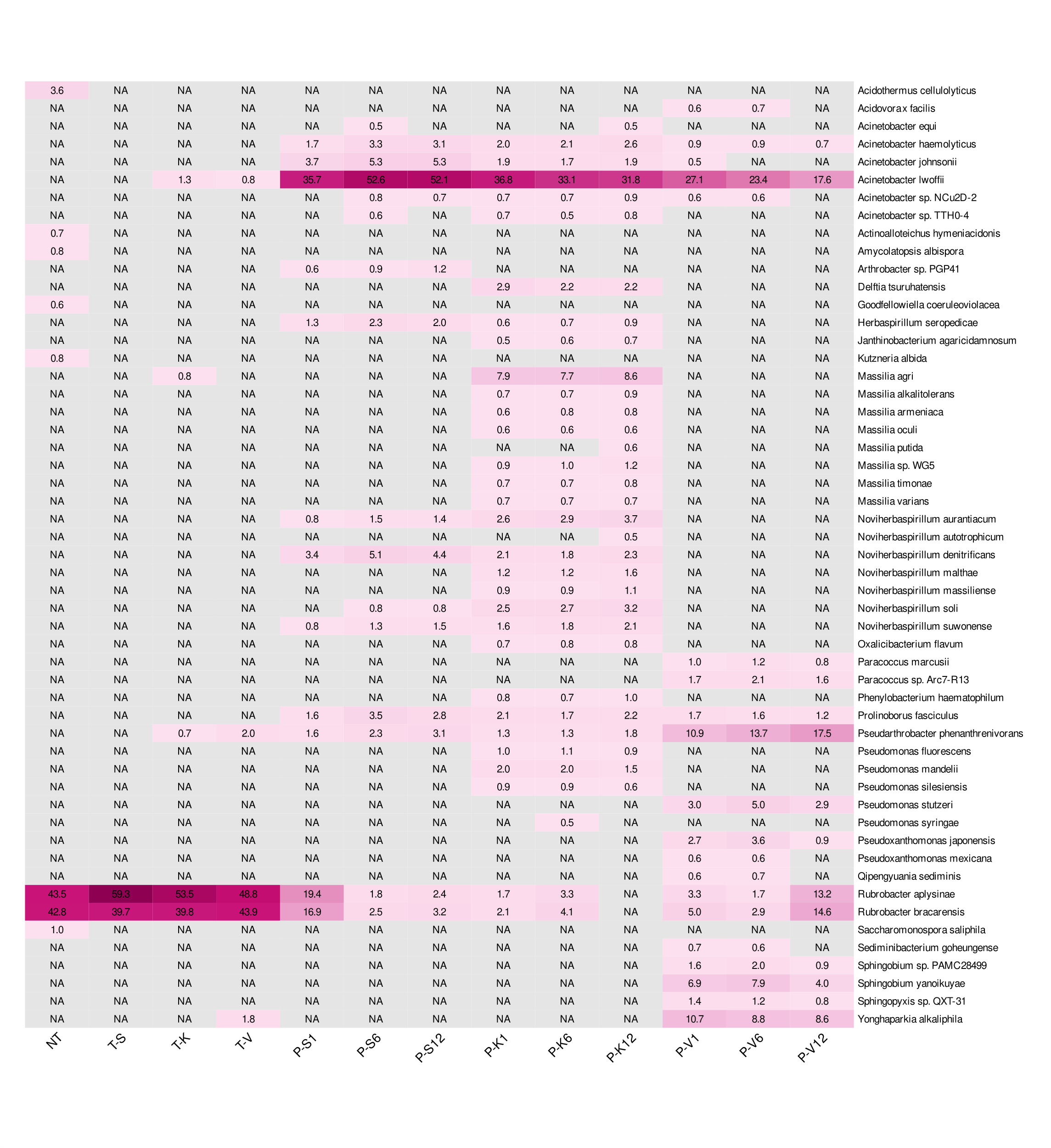


**Figure S4.** Heatmap showing the relative abundance in % of bacteria on species level (cut-off at 0.1%) for samples taken at the Charterhouse Mauerbach (M-4) during the application of the poultices (Poultice [P]; sepiolite [S]; kaolinite [K]; vermiculite [V]; non-treated [NT], after the treatment [T+S/K/V]) within the time intervals after one month [1m]; six months [6m] and 12 months [12m]. Colours correspond to the abundance values, the darker the colour, the higher the relative abundance. NA denotes measurements where the given species was not detected above the set cut-off value.


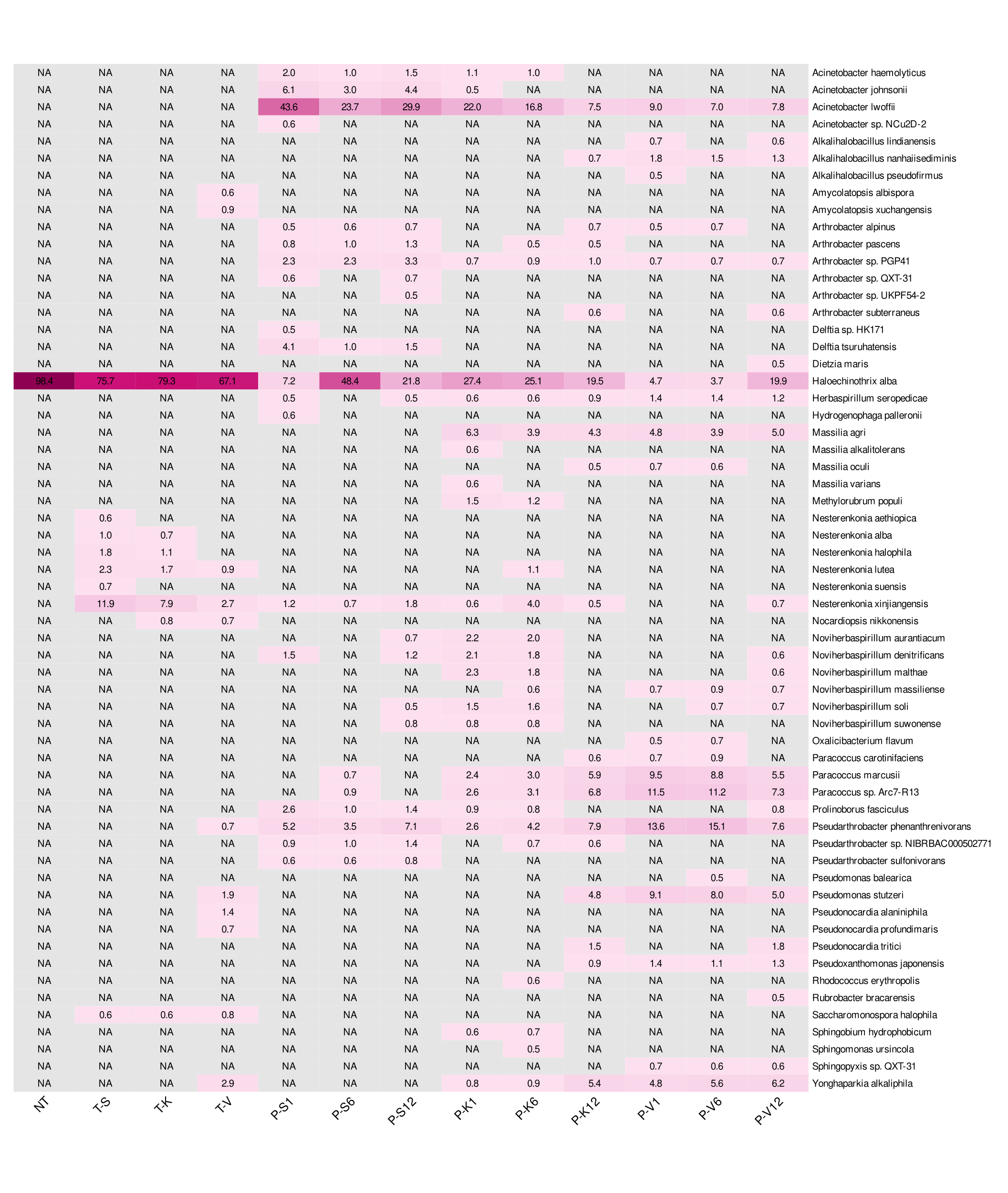


**a**

**Figure S5.** Heatmap showing the relative abundance in % of (a) bacteria and (b) archaea on species level (cut-off at 0.1%) for samples taken at the Charterhouse Mauerbach (M-6) during the application of the poultices (Poultice [P]; sepiolite [S]; kaolinite [K]; vermiculite [V]; non-treated [NT], after the treatment [T+S/K/V]) within the time intervals after one month [1m]; six months [6m] and 12 months [12m]. Colours correspond to the abundance values, the darker the colour, the higher the relative abundance. NA denotes measurements where the given species was not detected above the set cut-off value


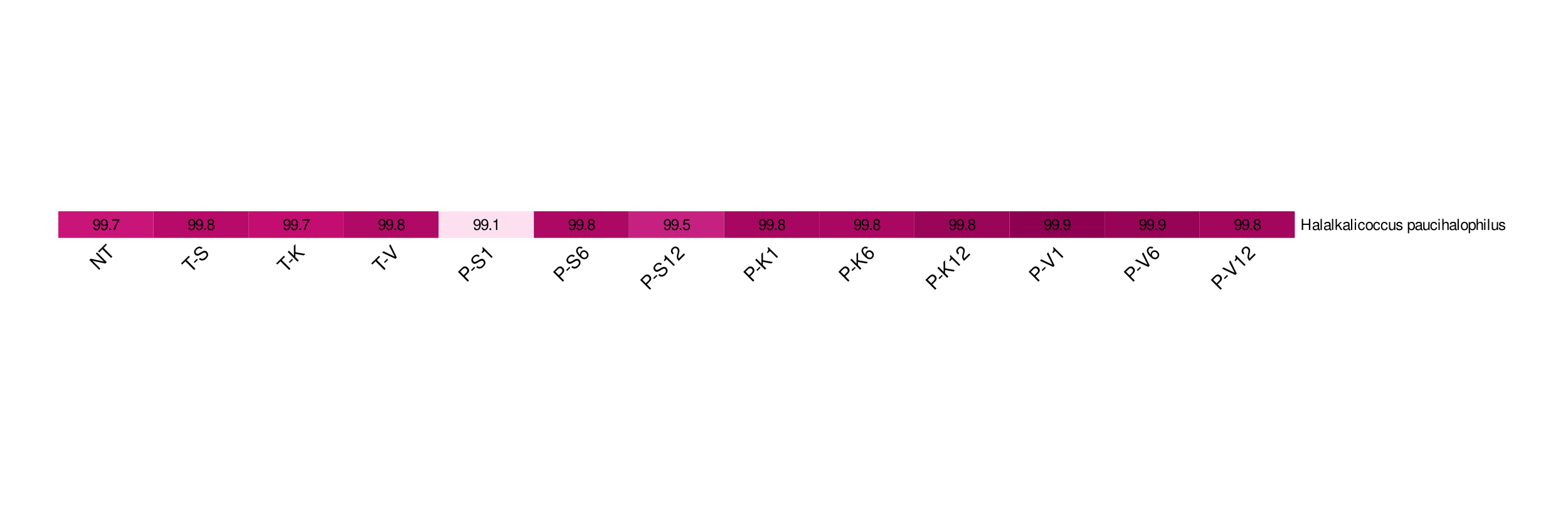


**b**

**Figure S5.** Heatmap showing the relative abundance in % of (a) bacteria and (b) archaea on species level (cut-off at 0.1%) for samples taken at the Charterhouse Mauerbach (M-6) during the application of the poultices (Poultice [P]; sepiolite [S]; kaolinite [K]; vermiculite [V]; non-treated [NT], after the treatment [T+S/K/V]) within the time intervals after one month [1m]; six months [6m] and 12 months [12m]. Colours correspond to the abundance values, the darker the colour, the higher the relative abundance. NA denotes measurements where the given species was not detected above the set cut-off value
